# Protective Antioxidant Potential of Argan Oil Versus Other Edible Oils in LPS-Challenged Mouse Heart and Kidney

**DOI:** 10.3390/ijms26178300

**Published:** 2025-08-27

**Authors:** Soufiane Rabbaa, Habiba Bouchab, Mounia Tahri-Joutey, Yassir Laaziouez, Youness Limami, Vivien Pires, Boubker Nasser, Pierre Andreoletti, Mustapha Cherkaoui-Malki, Riad El Kebbaj

**Affiliations:** 1 Sciences and Engineering of Biomedicals, Biophysics and Health Laboratory, Higher Institute of Health Sciences, Hassan First University, Settat 26000, Morocco; rabbaasoufian@gmail.com (S.R.); habibabouchab78@gmail.com (H.B.); y.laaziouez.doc@uhp.ac.ma (Y.L.); youness.limami@uhp.ac.ma (Y.L.); 2 Centre Des Sciences du Goût et de L’Alimentation (CSGA), Centre national de la recherche scientifique (CNRS), Institut national de recherche pour l’agriculture, l’alimentation et l’environnement (INRAE), Institut Agro, Université Bourgogne Europe, 21000 Dijon, France; mouniajoutey@gmail.com (M.T.-J.); vivien.pires@u-bourgogne.fr (V.P.); pierre.andreoletti@u-bourgogne.fr (P.A.); 3 Higher Institute of Nursing Professions and Technical Health (ISPITS), Errachidia 52000, Morocco; 4 Laboratory of Biochemistry, Neurosciences, Natural Resources and Environment, Faculty of Science and Technology, Hassan First University, Settat 26000, Morocco; boubker.nasser@uhp.ac.ma

**Keywords:** argan oil, olive oil, cactus seed oil, colza oil, oxidative stress, LPS, heart, kidney, SOD, catalase, GPx, GSH, MDA, z-score, clustering, Pearson correlation, PCA, FRAP, DPPH, ABTS, polyphenols

## Abstract

Oxidative stress plays a key role in tissue damage during inflammation, highlighting the need for effective antioxidant interventions. This study investigates the antioxidant potential of argan oil (AO)—obtained from *Argania spinosa (L.) Skeels* almonds—in comparison with olive oil (OO), cactus seed oil (CSO), and colza oil (CO). Quantitative analyses of total polyphenols and pigments—including chlorophylls, carotenoids, and xanthophylls—were conducted alongside antioxidant capacity assessments via DPPH, ABTS, and FRAP assays. The methanolic fraction consistently demonstrated the highest phenolic concentration and antioxidant efficacy across all oils. To establish in vivo relevance, a male C57BL/6J mouse model of acute oxidative stress was induced by lipopolysaccharide (LPS) administration. Pretreatment with oils significantly modulated key oxidative stress biomarkers—SOD, CAT, GPx activities, GSH levels, and lipid peroxidation (MDA)—in both heart and kidney. LPS challenge induced marked oxidative imbalance, notably increasing enzymatic activity and MDA levels, while depleting GSH in the heart and elevating it in the kidney. However, pretreatment with oils effectively restored redox homeostasis, with AO showing particularly potent effects and a stronger regulatory effect observed in the kidney. Hierarchical clustering of z-score-normalized heatmaps revealed distinct oxidative stress signatures, clearly separating LPS-treated heart and kidney tissues from other groups due to heightened oxidative markers. In contrast, oil-treated and oil-combined-with-LPS groups clustered closer to the control, underscoring the protective effect of oils against LPS-induced oxidative stress, with efficiency varying by oil type. Pearson correlation analysis, complemented by multivariate principal component analysis (PCA), further emphasized strong positive associations between antioxidant enzymes (SOD, CAT, GPx) and MDA levels, while GSH exhibited tissue-specific behavior—negatively correlated in the heart but positively in the kidney—highlighting divergent redox regulation between organs. Collectively, AO demonstrated robust cardioprotective and nephroprotective properties, supporting its potential as a natural dietary strategy against inflammation-induced oxidative stress.

## 1. Introduction

Oxygen (O_2_) is an essential molecule for the survival of all aerobic organisms. Often referred to as the Janus gas, molecular oxygen plays a dual role—while essential for cellular respiration, it also serves as a source of reactive oxygen species (ROS), including radical species like superoxide anion (O_2_^•−^) and non-radical species such as hydrogen peroxide (H_2_O_2_) [1]. These ROS can react with various biomolecules, such as lipids, proteins, and nucleic acids [2], with possible harmful repercussions in biological systems [1,3]. To counteract oxidative insults, organisms have developed a complex antioxidant defense system. This carries enzymatic antioxidants such as superoxide dismutase (SOD), glutathione peroxidase (GPx), and catalase (CAT), which constitute the first line of enzymatic antioxidant defense, as well as non-enzymatic metabolites like reduced glutathione (GSH) [4]. Subsequently, H_2_O_2_ is further decomposed into water through the enzymatic actions of GPx and CAT. Notably, the activity of GPx depends on the presence of GSH, which serves as a hydrogen donor [3]. In addition, GSH contributes to the scavenging of free radicals, reinforcing the cellular antioxidant capacity [5]. Interestingly, peroxisomes are unique organelles that house both ROS-generating oxidases and a range of antioxidant enzymes capable of neutralizing H_2_O_2_ and other harmful oxidative species [6]. The breakdown of H_2_O_2_ is primarily mediated by peroxisomal catalase [7]. Additional ROS-scavenging enzymes localized in peroxisomes include glutathione peroxidase (GPx), Cu/Zn superoxide dismutase (SOD), epoxide hydrolase, peroxiredoxin I, and peroxisomal membrane protein 20 [8]. However, when the formation of ROS overwhelms the antioxidant defenses, oxidative stress is triggered [3].

Among the agents known to induce oxidative stress, lipopolysaccharide (LPS)—a major endotoxin derived from Gram-negative bacteria—is widely used in experimental models [9,10,11,12]. Upon administration, LPS stimulates toll-like receptor 4 (TLR4), triggering a cascade of pro-inflammatory signaling pathways, including Nuclear factor kappa-light-chain-enhancer of activated B cells (NF-κB) activation and cytokine release [13]. This inflammatory surge is closely accompanied by excessive ROS generation from multiple sources—including nicotinamide adenine dinucleotide phosphate (NADPH) oxidases, mitochondria, and xanthine oxidase—leading to rapid depletion of antioxidant defenses and triggering oxidative stress [14]. Notably, the kinetics of LPS-induced responses are time-dependent and play a critical role in determining the nature and severity of oxidative responses [15,16]. Early time exposure of LPS has been shown to cause the initial signs of immune activation—such as reduced grip strength in mice—highlighting early functional decline and inflammatory signaling that often precedes overt oxidative injury [17]. However, prolonged exposure durations of LPS can lead to mitochondrial oxidative damage—including mitochondrial GSH depletion and mtDNA oxidation—in cardiac tissue [18] and are associated with sustained inflammation, marked by elevated myeloperoxidase (MPO) activity and neutrophil infiltration in pulmonary tissues, indicative of tissue-level oxidative damage and developing dysfunction [15]. Importantly, LPS has been shown to cause significant structural and functional damage in vital organs, particularly the heart and kidneys, where it disrupts redox balance, impairs mitochondrial function, and alters lipid metabolism [19,20,21,22]. These pathophysiological alterations contribute to organ dysfunction and are strongly associated with increased mortality, particularly in the context of human sepsis, as demonstrated in both clinical studies and experimental animal models [6,23]. Understanding the kinetics of LPS-induced oxidative stress is therefore essential for elucidating the pathophysiology of sepsis and for optimizing antioxidant therapeutic interventions.

Edible oils are rich sources of bioactive compounds currently recognized for their health-promoting effects against various pathologies through a large variety of action mechanisms, such as modulation of oxidative stress [24,25,26,27,28,29,30,31,32,33,34,35,36,37]. Among them, argan oil (AO), olive oil (OO), cactus seed oil (CSO), and colza oil (CO) stand out due to their distinctive compositions, characterized by high contents of unsaturated fatty acids, polyphenols, phytosterols (notably spinasterol and schottenol in AO vs. β-sitosterol in OO, CSO, and CO), pigments, and tocopherols [38,39,40]. AO is a traditional ingredient—for culinary, cosmetic, and medicinal uses—extracted from almonds of *Argania spinosa (L.) Skeels*, an endemic tree in Morocco [41]; it plays crucial agroforestry and socio-economic roles for the Amazigh residents in the southwestern region of Morocco [42,43]. In addition, olive and cactus seed oils are widely used in the Mediterranean diet [44,45], whereas CO is among the most commonly consumed oils in Europe [44]. Particularly, our research team has previously demonstrated that AO supplementation restored peroxisomal and mitochondrial function, improved the expression of nuclear receptors involved in fatty acid oxidation, and mitigated inflammation in the livers of LPS-challenged mice [9,11,46].

Clinical evidence supports the beneficial effects of these oils on cardiovascular and renal function in humans, particularly due to their content of bioactive compounds, which are known to exhibit antioxidant and anti-inflammatory properties. AO has been shown to significantly improve lipid profiles by lowering total cholesterol, LDL cholesterol, and triglycerides, while increasing HDL cholesterol levels [1,2]. These effects have been observed both in healthy individuals and in patients with type 2 diabetes and dyslipidemia, suggesting a cardioprotective potential and a reduction in LDL susceptibility to oxidation [47,48,49]. Similarly, olive oil—particularly in its extra virgin or high-polyphenol forms—has consistently demonstrated cardiovascular benefits, including improved lipid metabolism, lowered blood pressure, enhanced endothelial function, and a reduced incidence of major cardiovascular events. Moreover, long-term adherence to a Mediterranean diet rich in extra virgin olive oil has been associated with slower progression of renal dysfunction, especially in patients with coronary artery disease and type 2 diabetes [50,51,52,53,54,55]. Studies involving colza oil have also reported favorable effects on cardiovascular markers, such as increased apolipoprotein A-I levels and reduced visceral adiposity index in patients with type 2 diabetes. However, no significant changes in kidney-specific biomarkers were observed in overweight or obese individuals following moderate-term consumption [56,57,58]. Although large-scale clinical data on cactus seed oil are lacking, experimental studies using *Opuntia ficus-indica* cladode extracts have demonstrated significant diuretic and hypotensive effects in animal models, suggesting promising renal and cardiovascular benefits that warrant future human trials [59,60]. Despite these promising clinical outcomes, there is a clear need to investigate the antioxidant potential of these oils on cardiac and renal tissues. Notably, no comparative in vivo studies have yet explored their effects on antioxidant enzyme activities and oxidative stress markers altered by LPS in either the heart or kidneys.

In this context, the present study aimed to evaluate and compare, for the first time, the antioxidant capacities of the total oil, lipophilic, and methanolic fractions from AO, OO, CSO, and CO using DPPH, ABTS, and FRAP assays, along with the quantification of polyphenol and pigment contents. Furthermore, we investigated the protective effects of AO against acute (4 h) LPS-induced oxidative stress in murine heart and kidney tissues, with additional comparisons to OO, CSO, and CO. The analysis focused on five key oxidative stress parameters—SOD, GPx, CAT, GSH, and MDA—and included correlation and principal component analyses to identify oil-specific and tissue-specific protective patterns.

## 2. Results

To explore the antioxidant effect of AO versus OO, CSO, and CO, we first quantified their total chlorophyll, carotenoid, xanthophyll, and total phenolic content (TPC), and evaluated their radical scavenging capacities using ABTS, DPPH, and FRAP assays. Subsequently, we assessed their in vivo antioxidant effects by measuring the enzymatic activities of SOD, CAT, and GPx, as well as the levels of GSH and MDA, in the heart and kidney tissues of mice, using LPS as a stimulus of oxidative stress, thereby mimicking the inflammatory conditions observed in several chronic diseases. The heart and kidney were selected as target organs due to their central role in maintaining homeostasis, often referred to as the cardio-renal axis. This axis represents the bidirectional interaction between cardiac and renal functions, where dysfunction in one organ can directly affect the other. Both organs are particularly vulnerable to oxidative stress because of their high metabolic demands and continuous exposure to ROS. Studying them in parallel provides a better understanding of systemic oxidative disturbances and the potential protective role of dietary antioxidants.

### 2.1. Composition of Oils

Table 1 illustrates the chemical composition of AO compared to OO, CSO, and CO. The fatty acid profile of AO demonstrates a balanced distribution between oleic acid (43.52%) and linoleic acid (35.39%), whereas OO and CO show a marked predominance of oleic acid with 62.31% and 77.18%, respectively. However, CSO shows a predominance of linoleic acid (67.32%). Additionally, the palmitic acid content of AO (12.99%) is 2.15 times higher than that of OO (6.02%), and two times higher than that of CO (6.48%). AO also showed the highest content of stearic acid (6.71%) versus the other oils. In terms of minor antioxidant compounds, AO contained 0.05% stigmast-7-en-3-ol and 0.02% γ-tocopherol as the major tocopherol. However, OO was particularly rich in squalene, accounting for 28.27%, while AO contained only 0.33% of this compound.

### 2.2. Pigment Amounts and Total Polyphenol Content

**Figure 1:**
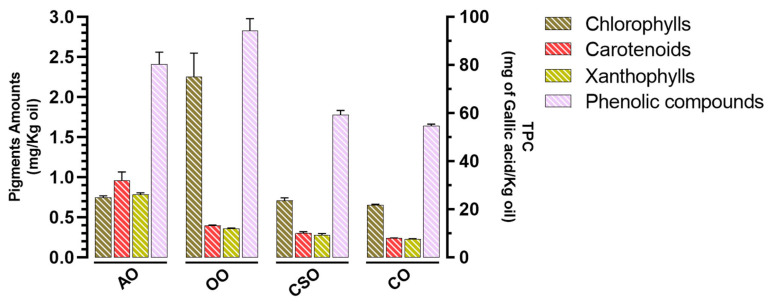
Total phenolic content (TPC) (mg of gallic acid/Kg oil), chlorophyll, carotenoid, and xanthophyll contents (mg/Kg oil) determined in argan oil (AO), olive oil (OO), cactus seed oil (CSO), and colza oil (CO). All values are means ± SD of data from three independent experiments (n = 3). Total phenolic content uses gallic acid as a reference molecule.

To further explore the natural antioxidant potential of AO, OO, CSO, and CO, we quantified their TPC and pigment amounts, including chlorophylls, carotenoids, and xanthophylls (Figure 1). Regarding chlorophyll content, OO exhibited the highest level (2.25 mg/kg), approximately three times higher than that of AO at 0.74 mg/kg. CSO and CO showed comparable chlorophyll levels of 0.70 and 0.65 mg/kg, respectively. In contrast, AO displayed the highest carotenoid content (0.96 mg/kg), which was about 2.5 times higher than OO (0.39 mg/kg), 3.2 times higher than CSO (0.30 mg/kg), and roughly four times that of CO (0.23 mg/kg). Similarly, AO had the greatest xanthophyll content (0.78 mg/kg), nearly 2.2 times higher than OO (0.35 mg/kg), 2.8 times higher than CSO (0.28 mg/kg), and 3.4 times higher than CO (0.22 mg/kg). Regarding TPC, expressed in mg GAE/Kg of oil, OO exhibited the highest level (94 mg/kg), followed by AO (80 mg/kg), CSO (59 mg/kg), and CO (55 mg/kg).

To gain deeper insight into the distribution of polyphenols in each vegetable oil, TPC was evaluated in the respective methanolic fraction (MF), lipophilic fraction (LF), and unfractionated or total fraction (TF) (Table 2). Quantitative analysis revealed notable differences in TPC among these fractions. In all oils, the highest polyphenol concentrations were consistently found in the MF, followed by the TF and LF. Among the MF, OO exhibited the highest polyphenol content, followed by AO, CSO, and CO. In contrast, both the lipophilic and total fractions showed lower polyphenol levels across all oils.

### 2.3. Antioxidant Activity of Methanolic, Lipophilic, and Total Fractions of Oils in Non-Cellular In Vitro Models

The antioxidant activities of the MF, LF, and TF of the oils were systematically analyzed using non-cellular in vitro assays, including ABTS, DPPH, and FRAP. The results revealed substantial variability not only between different oils but also among the fractions within each oil. This comparative analysis highlighted the distinct antioxidant potential of each fraction and shed light on the specific contribution of hydrophilic and lipophilic components to the overall antioxidant profile.

**Figure 2:**
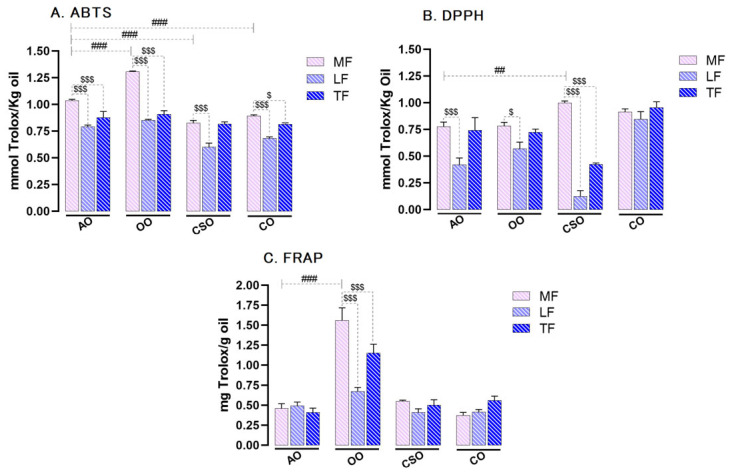
Antioxidant activities determined by ABTS (**A**), DPPH (**B**), and FRAP (**C**) of the methanolic fraction (MF), lipidic fraction (LF), and total fraction (TF) from argan oil (AO), olive oil (OO), cactus seed oil (CSO), and colza oil (CO). All values are means ± SD of data from three independent experiments (n = 3), and statistical significance of higher mean signal strength ($$$ *p* ≤ 0.001, $ *p* ≤ 0.05) is indicated compared to the MF of each oil, and (### *p* ≤ 0.001, ## *p* ≤ 0.01) is indicated compared to the MF of AO. Statistics were performed using two-way ANOVA followed by the Tukey test for multiple comparisons. Trolox was used as a reference molecule.

For the ABTS and DPPH assays, antioxidant activity was expressed both as percentage inhibition of pre-formed radicals (Table 2) and as mmol of Trolox equivalents per kilogram of oil (Figure 2). In the ABTS assay, inhibition percentages ranged from 46.46% ± 2.2 to 91.75% ± 0.42. The methanolic fractions demonstrated significantly stronger antioxidant activity than the lipophilic and total fractions. Notably, the MF of OO exhibited the highest scavenging activity (91.75% ± 0.42), followed by the MF of AO (74.4% ± 0.67).

Quantitatively, the MF of AO demonstrated an antioxidant capacity of 1.03 mmol TE/kg oil, which was significantly higher than its LF (0.79 mmol TE/kg; *p* ≤ 0.001) and TF (0.87 mmol TE/kg; *p* ≤ 0.001), representing increases of 1.3-fold and 1.18-fold, respectively. Compared to other oils, the MF of AO was 1.25-fold and 1.27-fold more potent than cactus seed oil (CSO; 0.82 mmol TE/kg; *p* ≤ 0.001) and corn oil (CO; 0.89 mmol TE/kg; *p* ≤ 0.001), respectively. The MF of OO showed even greater antioxidant activity (1.30 mmol TE/kg; *p* ≤ 0.001), approximately 1.26-fold higher than AO. According to the DPPH assay, inhibition percentages ranged from 15.66% ± 3.46 to 69.12% ± 3.51. Again, the MF of OO exhibited the highest scavenging capacity (69.12% ± 3.51). The MF of AO showed significant antioxidant activity (0.77 mmol TE/kg), 1.84-fold and 1.04-fold greater than its LF (0.42 mmol TE/kg; *p* ≤ 0.001) and TF, respectively, and was comparable to OO (0.78 mmol TE/kg). Interestingly, the MF of CSO demonstrated the highest DPPH activity (0.99 mmol TE/kg; *p* ≤ 0.001), about 1.28-fold higher than AO.

The FRAP assay, used to assess ferric reducing antioxidant power, showed that the MF and TF of OO had the highest activity, with 1.56 and 1.15 mg TE/g oil, respectively. Statistical analysis revealed a strong positive correlation between the TPC of the oil fractions and their antioxidant activities in all three assays (ABTS, DPPH, and FRAP), suggesting that the phenolic compounds play a central role in the radical scavenging and reducing capacities of these oils.

### 2.4. Effects of Argan Oil on LPS-Induced Heart and Kidney Oxidative Stress: In Vivo Approaches

#### 2.4.1. Effect of Argan Oil on the Regulation of Antioxidant Enzyme Activities in the Heart and Kidney

LPS is an acylated saccharolipid known for its ability to disrupt intracellular redox homeostasis and induce oxidative stress [9]. The cellular first line of defense against oxidative stress primarily consists of both enzymatic and non-enzymatic antioxidant systems, which act promptly to neutralize ROS and prevent oxidative damage. Key enzymes include superoxide dismutase (SOD), catalase (CAT), and glutathione peroxidase (GPx). Among them, SOD plays a pivotal role in ROS metabolism by catalyzing the dismutation of the superoxide anion (O_2_^•−^) to hydrogen peroxide (H_2_O_2_) and molecular oxygen (O_2_) [62].

**Figure 3:**
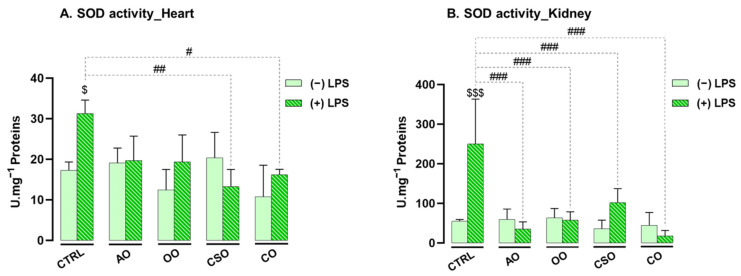
Effect of argan oil, cactus seed oil, olive oil, and colza oil treatments on SOD activity in the heart (**A**) and kidney (**B**). Male C57BL/6 mice received, for 28 days, a standard diet (control (CTRL)), a diet enriched with 6% (*w*/*w*) AO, CSO, OO, or CO, and an intravenous injection of LPS (100 μg) four hours antemortem. Heart and kidney homogenates were prepared as described in the Section 4. Results are expressed in UI·mg^−1^ = μmol of substrate transformed/minute/mg of protein. All values are means ± SD (n = 4–6), and statistical significance of higher mean signal strength (### *p* ≤ 0.001, ## *p* ≤ 0.01, # *p* ≤ 0.05) is indicated compared to LPS, and ($$$ *p* ≤ 0.001, $ *p* ≤ 0.05) is indicated compared to treatment with or without LPS administration. Statistics were performed using two-way ANOVA followed by the Tukey test for multiple comparisons.

At the end of the treatment period, SOD enzymatic activity was measured in heart and kidney homogenates from the different mouse groups. As shown in Figure 3, short-term exposure to LPS (5 mg/kg body weight) significantly increased SOD activity in both organs, with rises of +45% in the heart (*p* = 0.0353) and +78% in the kidney (*p* ≤ 0.001) compared to control mice. These increases reflect a compensatory antioxidant response triggered by LPS-induced oxidative stress.

Administration of the oils alone (AO, OO, CSO, or CO) did not significantly affect SOD activity in either organ, indicating no basal modulation of this enzyme under normal physiological conditions. In cardiac tissue, co-treatment with LPS and either AO (AO+LPS) or OO (OO+LPS) showed a non-significant downward trend in SOD activity, with reductions of −60% (*p* = 0.1920) and −57% (*p* = 0.1650), respectively. In contrast, co-treatment with CSO (CSO+LPS) or CO (CO+LPS) significantly attenuated the LPS-induced increase, reducing SOD activity by −48% (*p* = 0.0066) and −37% (*p* = 0.0342), respectively, bringing activity levels close to those of the control group. In renal tissue, co-supplementation of LPS with each of the four oils effectively counteracted the LPS-induced elevation in SOD activity. Reductions compared to the LPS group were as follows: AO+LPS (−75%, *p* ≤ 0.001), CO+LPS (−86%, *p* ≤ 0.001), CSO+LPS (−93%, *p* ≤ 0.001), and OO+LPS (−59%, *p* ≤ 0.001).

These findings underscore a tissue-specific antioxidant response and suggest that the tested oils modulate SOD activity in vivo, contributing to the re-establishment of redox homeostasis under short-term LPS-induced oxidative stress.

Following the evaluation of SOD activity, CAT enzymatic activity was measured in both cardiac and renal tissues from the different groups of mice. CAT is a key antioxidant enzyme predominantly localized in the peroxisomes of eukaryotic cells. As a typical catalase, it rapidly catalyzes the decomposition of H_2_O_2_ into water (H_2_O) and molecular oxygen (O_2_), thereby contributing to the regulation of oxidative stress [63]. Unlike manganese catalases or catalase-peroxidases primarily found in bacteria, typical catalase is widely expressed in animal tissues [63].

**Figure 4:**
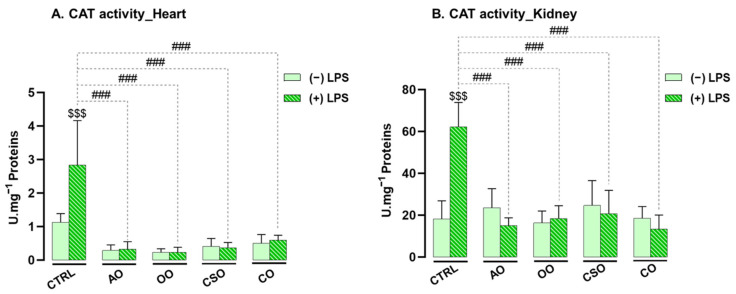
Effect of argan oil, cactus seed oil, olive oil, and colza oil treatments on CAT activity in the heart (**A**) and kidney (**B**). Male C57BL/6 mice received, for 28 days, a standard diet (control (CTRL)), a diet enriched with 6% (*w*/*w*) AO, CSO, OO, or CO, and an intravenous injection of LPS (100 μg) four hours antemortem. Heart and kidney homogenates were prepared as described in the Section 4. Results are expressed in UI·mg^−1^ = nmol of substrate transformed/minute/mg of protein. All values are means ± SD (n = 4–6), and statistical significance of higher mean signal strength (### *p* ≤ 0.001) is indicated compared to LPS, and ($$$ *p* ≤ 0.001) is indicated compared to treatment with or without LPS administration. Statistics were performed using two-way ANOVA followed by Tukey test for multiple comparisons.

In our study, CAT activity was specifically assessed in heart and kidney homogenates (Figure 4). A single intraperitoneal injection of LPS, administered 4 h prior to sacrifice, induced a significant increase in catalase activity in both organs: +71% in the kidney (*p* ≤ 0.001) and +60% in the heart (*p* ≤ 0.001), compared to control mice. This increase likely reflects an endogenous antioxidant response to acute inflammatory stress triggered by LPS.

In contrast, administration of the oils alone (AO, OO, CSO, CO) did not affect CAT activity in either organ, indicating no baseline effect under physiological conditions. However, co-treatment with oils and LPS markedly reduced the LPS-induced CAT overactivation. In cardiac tissue, CAT activity was significantly reduced by 88% with AO (*p* ≤ 0.001), 92% with OO (*p* ≤ 0.001), 79% with CSO (*p* ≤ 0.001), and 87% with CO (*p* ≤ 0.001), compared to the LPS group. Similarly, in the kidney, CAT activity was decreased by 75% with AO (*p* ≤ 0.001), 71% with OO (*p* ≤ 0.001), 61% with CSO (*p* ≤ 0.001), and 71% with CO (*p* ≤ 0.001).

These results demonstrate that all four vegetable oils effectively mitigated LPS-induced CAT overactivation. This supports their antioxidant potential in both cardiac and renal tissues and underscores their role in modulating enzymatic responses to oxidative stress.

**Figure 5:**
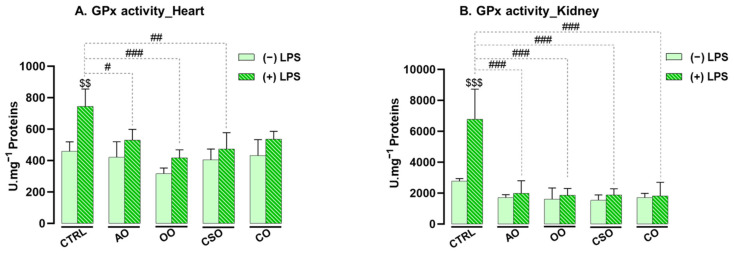
Effect of argan oil, cactus seed oil, olive oil, and colza oil treatments on GPx activity in the heart (**A**) and kidney (**B**). Male C57BL/6 mice received, for 28 days, a standard diet (control (CTRL)), a diet enriched with 6% (*w*/*w*) AO, CSO, OO, or CO, and an intravenous injection of LPS (100 μg) four hours antemortem. Results are expressed in UI·mg^−1^ = μmol of substrate transformed/minute/mg of protein. All values are means ± SD (n = 4–6). Statistical significance of higher mean signal strength (### *p* ≤ 0.001, ## *p* ≤ 0.01, # *p* ≤ 0.05) is indicated compared to LPS, and ($$$ *p* ≤ 0.001, $$ *p* ≤ 0.01) is indicated compared to the different treatments with or without LPS administration. Statistics were performed using two-way ANOVA followed by Tukey test for multiple comparisons.

GPx is a key antioxidant enzyme that uses reduced glutathione (GSH) as a cofactor to catalyze the conversion of H_2_O_2_ into water and oxygen, thereby protecting cells from oxidative damage [64]. Following the evaluation of SOD and CAT activities, GPx enzymatic activity was measured in cardiac and renal tissues (Figure 5).

LPS administration significantly increased GPx activity by +38% in the heart (*p* = 0.0042) and +59% in the kidney (*p* ≤ 0.001), reflecting an adaptive antioxidant response to LPS-induced oxidative stress.

In contrast, supplementation with the oils alone (AO, OO, CSO, or CO) did not affect GPx activity in either tissue. However, co-treatment with LPS and the oils significantly mitigated the LPS-induced increase in GPx activity. In cardiac tissue, co-administration with AO, OO, and CSO reduced GPx activity by 29% (*p* = 0.0336), 44% (*p* ≤ 0.001), and 36% (*p* = 0.0037), respectively, whereas CO induced a non-significant reduction of 28% (*p* = 0.0712). In contrast, in the kidney, all four oils markedly normalized GPx activity, with significant reductions of 71% for AO, 72% for OO, 72% for CSO, and 73% for CO (all *p* ≤ 0.001).

These findings underscore the protective roles of the oils in restoring LPS-induced oxidative stress, especially in renal tissue.

#### 2.4.2. Effect of Argan Oil on the Regulation of Oxidative Stress Biomarkers in the Heart and Kidney

In addition to enzymatic defenses, the non-enzymatic antioxidant defense system was assessed by quantifying reduced glutathione (GSH) in cardiac and renal tissues following supplementation and LPS challenge. GSH is a cytosolic tripeptide that plays a central role in maintaining redox homeostasis by directly neutralizing various ROS such as H_2_O_2_, O_2_^•−^, and OH^•^. It also contributes to metabolic detoxification and modulates immune responses [65].

**Figure 6:**
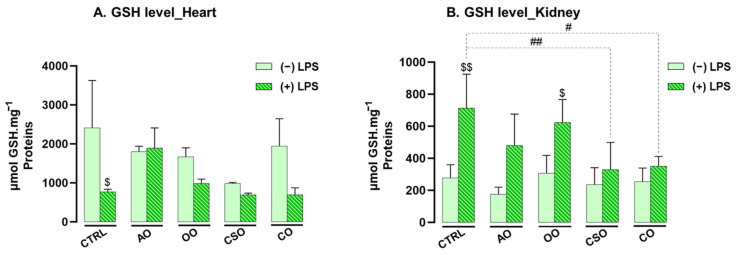
Effect of argan oil, cactus seed oil, olive oil, and colza oil treatments on GSH levels in the heart (**A**) and kidney (**B**). Male C57BL/6 mice received, for 28 days, a standard diet (control: CTRL), a diet enriched with 6% (*w*/*w*) AO, CSO, OO, or CO, and an intravenous injection of LPS (100 μg) four hours antemortem. All values are means ± SD (n = 4–6). Statistical significance of higher mean signal strength (## *p* ≤ 0.01, # *p* ≤ 0.05) is indicated compared to LPS, and ($$ *p* ≤ 0.01, $ *p* ≤ 0.05) is indicated compared to the different treatments with or without LPS administration. Statistics were performed using two-way ANOVA followed by the Tukey test for multiple comparisons.

As shown in Figure 6, acute LPS exposure led to a significant depletion in cardiac GSH (−113%, *p* = 0.0188), indicating oxidative stress, while a marked increase was observed in the kidney (+156%, *p* = 0.0020), suggesting a tissue-specific compensatory mechanism. Oil administration alone did not affect GSH levels in either organ.

However, co-treatment with oils and LPS elicited distinct modulatory effects. In the kidney, co-administration with CSO or CO significantly attenuated the LPS-induced increase in GSH levels by −54% (*p* = 0.0093) and −51% (*p* = 0.0250), respectively. In the heart, AO co-treatment tended to restore GSH levels (+125.75%), although the increase did not reach statistical significance.

These findings underscore the organ-specific regulation of redox status and suggest a more pronounced antioxidant effect of the tested oils in renal tissue.

**Figure 7:**
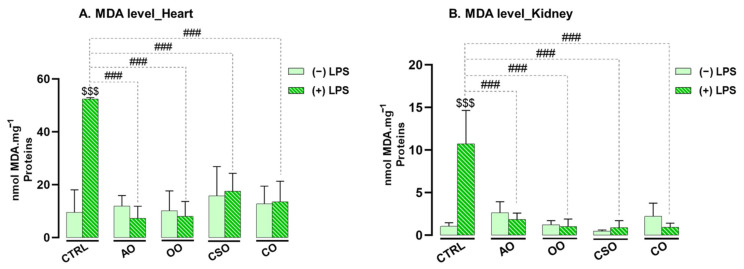
Effect of argan oil, cactus seed oil, olive oil, and colza oil treatments on MDA levels in the heart (**A**) and kidney (**B**). Male C57BL/6 mice received, for 28 days, a standard diet (control: CTRL), a diet enriched with 6% (*w*/*w*) AO, CSO, OO, or CO, and an intravenous injection of LPS (100 μg) four hours antemortem. All values are means ± SD (n = 4–6). Statistical significance of higher mean signal strength (### *p* ≤ 0.001) is indicated compared to LPS, and ($$$ *p* ≤ 0.001) is indicated compared to the different treatments with or without LPS administration. Statistics were performed using two-way ANOVA followed by the Tukey test for multiple comparisons.

Following the assessment of GSH levels, lipid peroxidation was evaluated by quantifying malondialdehyde (MDA) concentrations in both heart and kidney tissues. MDA is a well-established biomarker of lipid oxidative damage. As shown in Figure 7, LPS administration led to a significant increase in MDA levels in both organs, with elevations of +82% in the heart and +91% in the kidney (*p* ≤ 0.001 for both), indicating heightened lipid peroxidation.

However, while oil administration alone did not induce any significant changes in MDA levels—remaining comparable to those in control mice—co-treatment with oils in the presence of LPS significantly reduced MDA accumulation (*p* ≤ 0.001) in both organs. In the heart, MDA levels were reduced by 90% with AO, 91% with OO, 74% with CSO, and 83% with CO. Similarly, in the kidney, reductions were 83% with AO, 90% with OO, 73% with CSO, and 83% with CO.

These findings highlight the potent protective effects of the tested vegetable oils in preventing LPS-induced lipid peroxidation in both cardiac and renal tissues.

#### 2.4.3. Tissue-Specific Oxidative Stress Profiles: Heatmap Analysis, Pearson’s Correlation, and PCA

**Figure 8:**
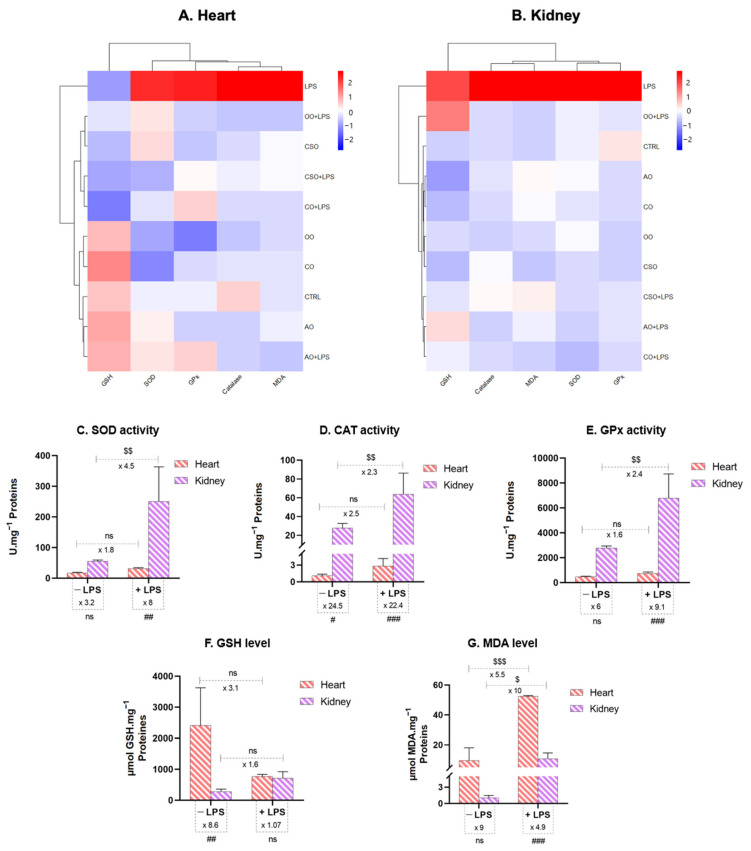
Tissue-specific comparison of oxidative stress markers in the heart and kidney under basal conditions and after LPS treatment. Heatmap showing the z-score-based variation of each oxidative stress parameter across the heart (**A**) and kidney (**B**). Red indicates an increase and blue a decrease relative to the overall mean. SOD (**C**), CAT (**D**), and GPx (**E**), as well as levels of GSH (**F**) and MDA (**G**), were measured in the heart and kidney tissues from control and LPS-treated mice. Bars represent mean ± SD (n = 4–6). Statistical significance of higher mean signal strength (### *p* ≤ 0.001, ## *p* ≤ 0.01, # *p* ≤ 0.05) is indicated compared to tissue, and ($$$ *p* ≤ 0.001, $$ *p* ≤ 0.01, $ *p* ≤ 0.05) is indicated compared to the LPS group. Statistics were performed using two-way ANOVA followed by the Tukey test for multiple comparisons.

Our data are summarized in a heatmap depicted in Figure 8, which illustrates z-score values of various oxidative stress markers—SOD, CAT, GPx, GSH, and MDA—in heart (Figure 8A) and kidney (Figure 8B) tissues across the different experimental conditions. This comparative approach enabled the identification of distinct, tissue-specific antioxidant response patterns. For the heart, the treatment conditions are clearly separated into three groups based on clustering. The first group only consists of LPS treatment, which is characterized by a lower GSH content and much higher enzyme activities compared to the z-score average (>2). The second group includes OO+LPS, CSO, CSO+LPS, and CO+LPS; these treatments are marked by both GSH content and enzymatic activities that are equivalent to or lower than the z-score average. The third group comprises OO, CO, CTRL, AO, and AO+LPS, all of which display equivalent higher GSH content and lower enzymatic activities relative to the z-score average. When clustering according to oxidative stress markers (columns), MDA and CAT exhibit the most similar patterns, followed by SOD and GPx, while GSH content stands out as the most distinct marker. Interestingly, the second group contains three LPS treatments—CO+LPS, OO+LPS, and CSO+LPS—and the third group contains three LPS-free treatments—OO, CO, and AO. Only CSO and AO+LPS treatments cluster in the second and third group, respectively. In contrast, the kidney displays a different pattern. Here, the LPS treatment results in higher levels than the z-score average for all markers. No clear clustering is observed for the other treatments. However, it is noteworthy that OO+LPS and AO+LPS show higher GSH content compared to the other treatments, although their levels do not reach those observed with LPS treatment.

Under baseline conditions (controls), enzymatic activities of SOD (Figure 8C), CAT (Figure 8D), and GPx (Figure 8E) were significantly higher in the kidney than in the heart, indicating a more robust intrinsic antioxidant defense in renal tissue. Following LPS administration, oxidative stress was significantly induced in both organs, as evidenced by the hyperactivation of SOD, CAT, and GPx. This response was more pronounced in the kidney, suggesting a stronger compensatory enzymatic response compared to the heart.

In contrast, basal levels of the non-enzymatic antioxidant GSH (Figure 8F) and the lipid peroxidation marker MDA (Figure 8G) were higher in the heart. After acute LPS exposure, GSH levels significantly declined in the heart, reflecting rapid depletion due to ROS neutralization. A significant depletion of GSH (Figure 8F) was observed in the heart, reflecting its rapid consumption in neutralizing excessive ROS. Conversely, the kidney showed a marked increase in GSH, possibly reflecting an upregulated synthesis or recycling mechanism as part of its adaptive response. MDA levels increased significantly in both tissues, though the heart exhibited a more substantial elevation, indicating greater vulnerability to lipid peroxidation. These findings, clearly depicted in the z-score heatmap, highlight a tissue-specific oxidative stress profile: the kidney possesses a more resilient enzymatic antioxidant capacity, while the heart demonstrates greater susceptibility, particularly in terms of non-enzymatic antioxidant depletion and lipid peroxidation. This differential response underscores the need for organ-targeted strategies in managing oxidative stress-related pathologies.

**Figure 9:**
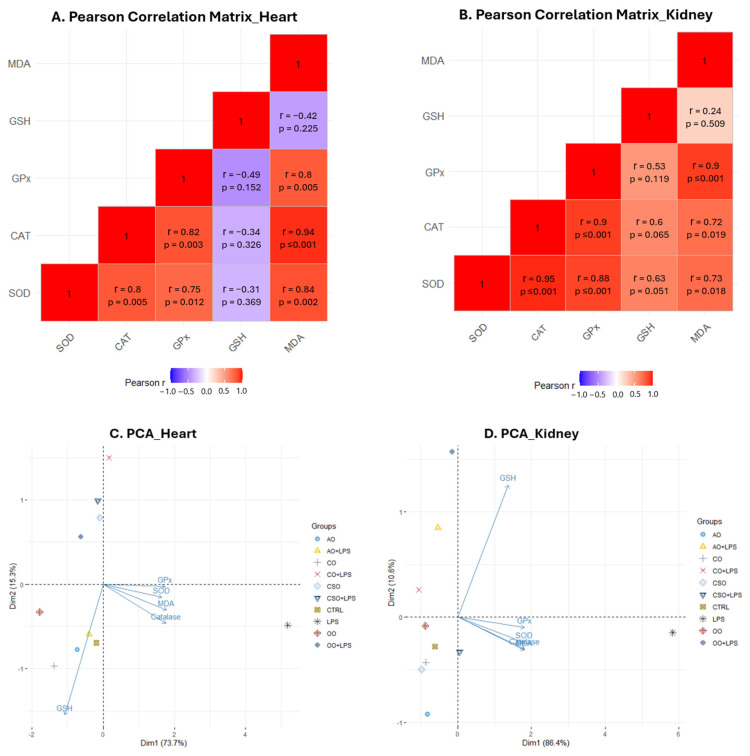
Pearson correlation analysis and principal component analysis (PCA) of oxidative stress markers in the heart and kidney tissues (n = 4–6). (**A**,**B**) Pearson correlation matrices of oxidative stress markers in the heart (**A**) and kidney (**B**). Pearson correlation coefficients were calculated between enzymatic antioxidants (SOD, CAT, GPx), the non-enzymatic antioxidant (GSH), and the lipid peroxidation marker (MDA). The color scale indicates the strength and direction of correlations (blue: negative, red: positive). (**C**,**D**) Principal Component Analysis (PCA) biplots of oxidative stress parameters in the heart (**C**) and kidney (**D**). The axes represent the first two principal components (Dim1 and Dim2), capturing most of the variance. The length and direction of the arrows reflect the contribution (cos^2^) and correlation of each variable to the principal components.

Pearson correlation analysis was performed to explore the relationships among oxidative stress parameters (SOD, catalase, GPx, GSH, and MDA) in the heart and kidney tissues (Supplementary Table S1, Figure 9A,B), revealing distinct patterns in each organ.

In the heart, strong and significant positive correlations were observed among the antioxidant enzymes:

- CAT and GPx (r = 0.83; *p* = 0.003),

- SOD and CAT (r = 0.80; *p* = 0.005),

- SOD and GPx (r = 0.75; *p* = 0.012).

These enzymes also correlated positively with MDA levels:

- MDA and CAT (r = 0.94; *p* < 0.001),

- MDA and SOD (r = 0.84; *p* = 0.002),

- MDA and GPx (r = 0.80; *p* = 0.005).

These results suggest that oxidative damage, as reflected by MDA accumulation, increases in parallel with enzymatic antioxidant activity, likely as a compensatory mechanism.

Conversely, GSH levels in the heart showed non-significant negative correlations with SOD (r = −0.32), CAT (r = −0.35), GPx (r = −0.49), and MDA (r = −0.42), suggesting an inverse relationship between enzymatic and non-enzymatic antioxidant responses in this tissue.

In the kidney, correlations between enzymatic antioxidants were even stronger and highly significant:

- SOD and CAT (r = 0.95; *p* < 0.001),

- CAT and GPx (r = 0.90; *p* < 0.001),

- SOD and GPx (r = 0.88; *p* = 0.001).

MDA levels also showed positive and significant correlations with these enzymes:

- MDA and GPx (r = 0.90; *p* < 0.001),

- MDA and SOD (r = 0.72; *p* = 0.018),

- MDA and CAT (r = 0.72; *p* = 0.019).

These results suggest that LPS-induced oxidative stress elicits a more coordinated and efficient enzymatic antioxidant response in the kidney. Meanwhile, the non-enzymatic defense mechanism, as represented by GSH, may be governed by different regulatory dynamics.

Furthermore, a multivariate analysis via PCA plot (Figure 9C,D) was performed to explore the overall variations in oxidative stress parameters under different treatments, which revealed a clear separation between the control and LPS-treated mice, indicating that LPS significantly altered the oxidative stress profile in both heart and kidney tissues.

In the cardiac tissue (Figure 9C), the first two principal components explained 89.0% of the total variance (PC1: 73.7%, PC2: 15.3%). PC1 was mainly driven by strong positive contributions from GPx, SOD, CAT, and MDA, which were tightly clustered and positively correlated, suggesting that lipid peroxidation and enzymatic antioxidant responses were the main drivers of variability. This confirms the prominent oxidative stress induced by LPS. On the other hand, GSH was oriented in the opposite direction, contributing primarily to PC2 and negatively associated with the other oxidative stress parameters. This inverse relationship suggests a potential antagonistic behavior of GSH compared to both oxidative stress levels (MDA) and enzymatic antioxidant activities. Elevated GSH levels might reflect a more effective redox buffering capacity, possibly reducing the need for upregulation of enzymatic defenses. In contrast, higher MDA levels were associated with increased enzymatic activity, indicating a compensatory response to oxidative damage.

Notably, mice co-treated with natural oils (AO, OO, CSO, and CO) clustered separately from the LPS group, with the AO co-treatment appearing closest to the control group. This distribution suggests a partial, and in the case of AO, potentially full restoration of redox balance. This trend highlights the special antioxidant potential of AO in counteracting LPS-induced oxidative damage in the heart.

In the kidney (Figure 9D), the PCA explained 97.0% of the total variance (PC1: 86.4%, PC2: 10.6%). Similar to the heart, SOD, GPx, CAT, and MDA were grouped together and positively correlated, forming a compact cluster along PC1. GSH again appeared isolated, contributing strongly in a direction distinct from the antioxidant enzymes and MDA. This pattern reinforces the protective and independent role of GSH in renal tissue. The close proximity of antioxidant enzyme activities and MDA suggests a highly coordinated oxidative stress response, where increased lipid peroxidation is paralleled by upregulation of antioxidant enzyme responses. However, treatments showed distinct clustering, particularly in co-treatment with AO, OO, and CO, which were positioned away from both the control and LPS groups, suggesting a unique modulatory effect on the kidney’s oxidative environment.

Overall, the PCA highlights dual antioxidant strategies: a rapid enzymatic response to oxidative challenge and a basal GSH-dependent buffering system. However, our findings confirm that multivariate analysis via PCA can efficiently capture complex biochemical alterations and offer complementary insights to pairwise correlation matrices. Moreover, PCA facilitates visualization of global trends and treatment effects that may not be evident in univariate or bivariate analyses.

## 3. Discussion

Natural oils have long been the main sources of remedies in human history. The unique composition of AO significantly underpins its antioxidant potential compared to other vegetable oils, including OO, CSO, and CO. Particularly, AO exhibits a balanced fatty acid profile, with oleic acid (43.5%) and linoleic acid (35.4%), unlike OO and CO, which are predominantly rich in oleic acid (62.3% and 77.2%, respectively), and CSO, which is rich in linoleic acid (67.3%). This balanced fatty acid ratio in AO aligns with previous literature reports [11,38,66,67] and is known for its beneficial effects in modulating oxidative stress and inflammation [68,69,70,71]. Moreover, the tocopherol profile of AO, dominated by γ-tocopherol as documented in prior studies [11,31], contributes substantially to its antioxidant efficacy due to the potent free radical scavenging activity of this isoform [72,73]. In addition to fatty acids and tocopherols, the antioxidant capacity of oils is strongly supported by their polyphenolic and pigment content, which are well established for their roles in free radical neutralization and mitigation of oxidative damage [40,74]. In the present study, significant differences were observed in total phenolic content (TPC) and pigment composition among the tested vegetable oils and across their respective methanolic (MF), lipophilic (LF), and total (TF) fractions. The variability in pigment content is likely influenced by several factors, including seed roasting, extraction temperature and duration, processing method, and storage conditions [75]. Likewise, TPC variability may be driven by the fruit ripeness, storage duration prior to oil extraction, harvest season, growth stage of the plant, and its genetic background [76,77,78].

As expected, the MF showed the highest polyphenol content of all oils, underscoring the hydrophilic nature of most phenolic compounds. Notably, the MF of OO and AO exhibited the greatest concentrations of polyphenols, followed by CSO and CO. These results are consistent with previous studies reporting that OO and AO are naturally rich in hydrophilic phenols such as ferulic acid, vanillic acid, and synergic acid [79,80,81,82].

Pigment analysis also revealed composition diversity. Chlorophylls were predominantly present in OO, while AO, CSO, and CO exhibited moderate amounts, as previously reported in the literature [83]. However, the relative abundance of carotenoids and xanthophylls in AO likely contributes to its antioxidant capacity, as these pigments are known to quench singlet oxygen and prevent lipid peroxidation [84,85].

These compositional differences were reflected in the antioxidant activity assays. Both DPPH and ABTS radical scavenging tests showed that the MF fractions had the highest activity, followed by TF and LF. In the ABTS assay, the MF of OO had the highest activity, followed closely by the MF of AO, corroborating earlier findings [83]. These results support the role of phenolic compounds in the oils’ antioxidant mechanism, especially since ABTS can react with both hydrophilic and lipophilic antioxidants. Of note, DPPH inhibition values ranged from 15.7% to 71.9%, with the MF of CSO exhibiting the highest activity, highlighting its potential for effective radical scavenging [86,87]. In the FRAP assay, which evaluates ferric reducing power, strong antioxidant was again observed in the MF and TF of OO, whereas LF showed significantly lower capacity. Overall, strong positive correlations were observed between TPC and antioxidant activity across all assays (DPPH, ABTS, FRAP), reaffirming that polyphenols are the key contributors—consistent with prior findings [83].

In light of these findings, and in order to better understand the source of antioxidant effects, we conducted a complementary in vitro assessment comparing the antioxidant potential of the MF, LF, and TF of each oil. As expected, the methanolic fractions—rich in polyphenols—exhibited the highest antioxidant activity in radical scavenging assays. However, the lipophilic fractions, which represent approximately 99% of the total oil mass, also demonstrated notable antioxidant potential, particularly in the ABTS and FRAP assays. These results highlight the relevance of including fatty acid profiling in antioxidant characterization, not solely for nutritional purposes, but also to better understand the contribution of the lipophilic fraction to the overall antioxidant capacity.

Ultimately, the in vivo experiments were performed using the total oils, allowing us to evaluate the potential synergistic interactions between saponifiable and unsaponifiable components, reflecting the complexity and real-life bioactivity of the oils. This integrative approach provides a realistic view of the oils’ biological effects.

The in vivo study was carried out in C57BL/6J mice subjected to acute oxidative stress induced by LPS administration, delivered intraperitoneally 4 h prior to euthanasia. LPS, a major component of Gram-negative bacterial membranes, is well known to induce systemic inflammation and oxidative stress, impairing multiple organs [12]. Our model aimed to mimic oxidative stress challenge to evaluate the oils’ potential protective roles in cardiac and renal tissues—two organs commonly affected by systemic oxidative insults.

We focused on classical oxidative stress markers: enzymatic antioxidants (SOD, CAT, and GPx), the non-enzymatic antioxidant (GSH), and the lipid peroxidation marker (MDA). To our knowledge, this is the first study comparing the potential protective effects of AO against OO, CSO, and CO in the heart and kidney under LPS-induced oxidative stress. We used the LPS serotype *Escherichia coli* O111:B4, consistent with several previous studies [9,10,11,46].

Our results highlight that LPS administration triggered a marked oxidative imbalance in both tissues, characterized by increased activities of SOD, CAT, and GPx, elevated MDA levels, and altered GSH content. These findings confirm the pro-oxidant nature of LPS and align with earlier reports of oxidative stress in the liver and brain following endotoxin exposure [9,11].

The upregulation of antioxidant enzyme activities reflects an adaptive response to counteract ROS accumulation, with more a pronounced effect observed in the kidney, likely reflecting its higher baseline antioxidant enzyme levels compared to the heart. The non-enzymatic defense via GSH displayed tissue-specific patterns: the heart showed a marked depletion of GSH, reflecting high consumption under oxidative stress, whereas the kidney showed increased GSH levels possibly due to enhanced synthesis or recycling mechanisms [88]. The reduction of GSH in the heart following LPS exposure aligns with findings by Suliman et al., who reported a decreased GSH/GSSG ratio after short-term LPS treatment (6 h) in cardiac tissue, indicating oxidative imbalance [18]. The cardiac GSH redox balance is particularly vulnerable under pathological conditions, rendering cardiomyocytes highly susceptible to oxidative damage [89]. Numerous studies have linked myocardial injury to early impairments in GSH synthesis and recycling [88]. In contrast, the kidney’s robust GSH response may stem from its physiological dependence on GSH, particularly in proximal tubular cells, which are metabolically active and prone to oxidative injury. Although around 80% of plasma GSH is reabsorbed during renal filtration—primarily in the proximal tubules [88,90]—the kidney also possesses the capacity to synthesize its own GSH, supporting its antioxidant resilience. Nevertheless, it was shown that in healthy murine tissues such as the heart and kidney of C57BL/6J mice, GSH levels are approximately tenfold higher than their oxidized form (GSSG) levels, suggesting that changes in GSH alone can still reflect oxidative imbalance under stress conditions [91,92].

Furthermore, MDA levels rose significantly in both organs but the increase was more prominent in the heart, suggesting a higher vulnerability to lipid peroxidation [93]. Importantly, treatment with vegetable oils alone did not affect oxidative stress parameters, indicating their safety and lack of pro-oxidant effects under basal conditions. However, when co-administered with LPS, all four oils significantly attenuated the LPS-induced increase in antioxidant enzyme activities and MDA levels, while partially restoring GSH content, particularly in the renal tissue. These findings support the oils’ protective roles in redox regulation during inflammatory stress.

The protective mechanism may involve activation of the nuclear factor erythroid-2-related factor 2 (Nrf2) transcription factor signaling pathway, a key regulator of antioxidant defense [1,24,31,94]. Under oxidative stress, ROS oxidize cysteine residues on Keap1 (Kelch-like ECH-associated protein 1), impairing its binding to Nrf2. This enables Nrf2 to translocate into the nucleus, where it dimerizes with small musculoaponeurotic fibrosarcoma (Maf) proteins and binds to antioxidant response elements (AREs), promoting the transcription of genes involved in antioxidant defenses [95,96,97]. Forkhead box O (FoxO) transcription factors also exert antioxidant effects by upregulating enzymes such as SOD and catalase. Additionally, FoxO contributes to GSH-dependent detoxification by enhancing the transcription of GPx1 [98].

The antioxidant properties of AO, OO, CSO, and CO probably stem from their specific compositions of fatty acids, tocopherols, and polyphenols [44,82], which may contribute to this compensatory mechanism by modulating enzymatic activity and supporting tissue repair during oxidative injury.

Hierarchical clustering analysis of z-score-normalized heatmaps revealed distinct oxidative stress signatures, clearly separating LPS-treated heart and kidney tissues from other groups due to heightened oxidative markers. Correlation analyses revealed strong positive associations between enzymatic antioxidants (SOD, CAT, GPx) and MDA in both tissues, reflecting that these enzymes are upregulated in response to oxidative damage. In contrast, GSH showed weak and negative correlations with enzymatic markers and MDA, particularly in the heart, pointing to its distinct, possibly earlier, role in the antioxidant defense hierarchy. The PCA results revealed distinct yet comparable oxidative stress profiles between heart and kidney tissues, suggesting tissue-specific regulatory mechanisms of antioxidant defense. The positive correlation between MDA and enzymatic antioxidant activities in both tissues suggests a feedback mechanism where elevated oxidative stress induces enzymatic antioxidant responses. This is consistent with a previous clinical study where MDA levels were significantly accompanied by elevated activities of SOD, GPx, and CAT, indicating an adaptive enzymatic antioxidant response to cellular oxidative insult [99]. In contrast, GSH appeared to act antagonistically and independently of enzymatic responses and MDA levels, supporting its well-established role as a primary intracellular redox buffer. Interestingly, the heart displayed a slightly less compact clustering of enzymatic variables and a more prominent role of GSH on PC2. This could reflect the heart’s higher vulnerability to oxidative injury due to its continuous aerobic metabolism and high mitochondrial content [100,101]. Overall, these PCA results underscore the complex interplay between oxidative damage and antioxidant systems and suggest that GSH operates as an early protective factor, while enzymatic responses represent adaptive mechanisms to rising oxidative pressure. The tissue-specific patterns observed here align with previous findings showing organ-dependent antioxidant regulation and susceptibility to oxidative stress.

Our study significantly adds to the existing body of knowledge by providing, for the first time, a direct comparative evaluation of the antioxidant and protective effects of four distinct edible oils—AO, OO, CSO, and CO—on oxidative stress induced by short-term LPS exposure. Importantly, this investigation was conducted in two metabolically and functionally distinct organs, the heart and the kidney, which are known to differ in their baseline antioxidant capacity and susceptibility to oxidative damage. This dual-organ approach allowed us to highlight tissue-specific antioxidant signatures, further supported by hierarchical clustering and correlation analyses. These findings not only confirm the antioxidant potential of argan oil but also position it within a broader nutritional and functional context compared to other widely consumed oils.

Despite the strengths of this study, certain limitations should be acknowledged. The number of animals used was minimized in accordance with ethical guidelines. Moreover, although the mouse model is widely accepted for studying oxidative stress, it does not fully reproduce human physiology, and caution is needed when extrapolating the findings to humans. Additionally, although our chemical-based antioxidant assays provided an initial comparison of the oils’ radical scavenging activity, they do not fully represent their biological behavior. Future studies should integrate cell-based assays to better investigate both direct and indirect antioxidant mechanisms and support mechanistic insights into oil bioactivity. Furthermore, the anti-inflammatory effects of the oils in our model remain to be fully elucidated and warrant further investigation. At the molecular level, although we evaluated multiple antioxidant parameters (SOD, CAT, GPx, GSH, and MDA) in heart and kidney tissues, the measurement of oxidized glutathione (GSSG) would have provided a more comprehensive understanding of the redox balance, particularly the GSH/GSSG ratio as a redox biomarker. Moreover, tissue-specific transcriptome profiling has shown differential regulation of SOD isoforms, depending on the organ and the oxidative context [102]. These points highlight the need for deeper molecular analyses in future investigations.

## 4. Materials and Methods

### 4.1. Chemicals and Reagents

All chemical products were purchased from Sigma-Aldrich (Saint-Quentin-Fallavier, France); the Pierce^TM^ BCA Protein Assay Kit was purchased from ThermoFisher Scientific (Illkirch, France); and the Potter Elvehjem homogenizer was purchased from Dominique Dutscher (Issy-les-Moulineaux, France).

### 4.2. Origin and Extraction of Oils

The AO was obtained from the company EFAS (Agadir, Morocco). The extraction was performed in five different steps: pulping the fruit, crushing the hull, and roasting the almond were performed manually, while grinding roasted almonds was performed by a mechanical press. OO was collected from the modern cooperative AZZABA (Sefrou, Morocco). It was extracted mechanically by crushing fruits and extracting the juice. CSO was obtained from the Cooperative of Sabbar Rhamna (Skhour Rhamna, Morocco). Seeds were separated from the juice using a domestic extractor (Philips Viva HR1832/00, Mumbai, India). After juice separation, the seeds were thoroughly washed with water, air-dried, and the oil was extracted by cold pressing using a mechanical expeller (Longer Machinery, LGYL-80A, Henan, China). CO was commercially obtained from a local supermarket. For the four oils, aliquots were kept under inert conditions and stored in the dark at 4 °C to prevent oxidation.

To characterize the volatile compounds, present in the oils, each sample oil was solubilized in chloroform prior to gas chromatography–mass spectrometry (GC-MS) analysis. GC-MS was performed using an Agilent 7890A Series instrument (Agilent Technologies, Santa Clara, CA, USA) equipped with a multimode injector and a 123-BD11 column (15 m × 320 μm × 0.1 μm). A 4 μL aliquot of each chloroform-solubilized extract was injected in split mode (1:4) using helium as the carrier gas at a flow rate of 3 mL/min. The ion source and quadrupole temperatures were maintained at 230 °C and 150 °C, respectively. The oven temperature was programmed from 30 °C to 360 °C. Compound identification was based on matching spectral data against the NIST 2014 MS Library (Gaithersburg, MD, USA). The results of oils quality indices are listed in Table 3.

### 4.3. Oils Characterization

#### 4.3.1. Determination of Chlorophyll, Carotenoid, and Xanthophyll Contents

Chlorophyll, carotenoid, and their derivative xanthophyll were determined spectrophotometrically at 670, 470, and 450 nm, respectively, by dissolving 92 mg of each oil in a final volume of 1 mL of cyclohexane [104]. Specific extinction coefficients were used: ε = 613 for pheophytin (according to chlorophyll fraction) and ε = 2000 for lutein (according to carotenoid and xanthophyll fraction).

#### 4.3.2. Extraction of Methanolic and Lipophilic Fractions of Oils

The oils were subjected to a liquid–liquid extraction to separate the polar and non-polar fractions, following the method described by Espín et al. (2000) [105]. Two g of each oil were dissolved in 1 mL of n-hexane and mixed vigorously. To extract the polar fraction, 2 mL of methanol/water (80:20, *v*/*v*) were added to the solution to initiate the extraction. The mixture was centrifuged at 2800× *g* for 5 min. The lower phase, corresponding to the methanolic fraction, contains the polar phenolic compounds, while the upper phase, corresponding to the lipophilic fraction, contains the non-polar components. An aliquot of unfractionated oil was also included in the analysis for comparison with the two separate fractions.

#### 4.3.3. Determination of Total Phenolics Contents

The TPC of the oils and their fractions was determined using the Folin–Ciocalteu colorimetric method as described by [106]. A total of 100 µL of each sample was mixed with 900 µL of diluted Folin–Ciocalteu reagent (1:10 dilution with distilled water). After 5 min of incubation in the dark, 750 µL of sodium carbonate (6%) were added. The reaction mixture was vigorously shaken and incubated at 45 °C for 15 min in the dark. Absorbance was measured spectrophotometrically at 765 nm. Results were expressed as milligrams of gallic acid equivalents per grams of oil (mg GAE/Kg oil).

#### 4.3.4. Antioxidant Activities of Oils

The antioxidant capacities of vegetable oils (AO, OO, CSO, and CO) and their fractions were assessed using three assays: ABTS radical scavenging, DPPH radical scavenging, and ferric reducing antioxidant power (FRAP).

For the ABTS assay [107], the ABTS radical cation was generated by reacting 7 mM ABTS with 2.5 mM potassium persulfate in phosphate buffer (5 mM, pH 7.4), followed by incubation in the dark for 16 h. The ABTS solution was then diluted with ethanol to an absorbance of 0.7 ± 0.02 at 734 nm. A volume of 40 µL of oil sample was mixed with 160 µL of ABTS, incubated for 10 min in the dark, and the absorbance was measured at 734 nm. All values were expressed as percentages of inhibition and millimoles of Trolox equivalents per kilogram of oil (mmol Trolox/Kg oil).

The DPPH assay was based on the ability of antioxidants to reduce the stable free radical DPPH (2,2′-diphenyl-1-picrylhydrazyl) by hydrogen atom transfer [108]. A total of 40 µL of oil sample was mixed with 240 µL of 0.2 mM DPPH solution prepared in methanol. After 30 min of incubation in the dark at room temperature, the absorbance was recorded at 517 nm. All values were expressed as percentages of inhibition and millimoles of Trolox equivalents per kilogram of oil (mmol Trolox/Kg oil).

For the FRAP assay, the ferric reducing antioxidant power was determined following the method of Oyaizu (1986) [109], which evaluates the reduction of the Fe^3+^-TPTZ complex to the blue-green Fe^2+^-TPTZ form. Briefly, 100 µL of sample was mixed with 250 µL of phosphate buffer (0.2 M, pH 6.6) and 250 µL of potassium ferricyanide (1%). After mixing, the reaction was incubated at 50 °C for 20 min. The reaction was stopped by adding 250 µL of 10% trichloroacetic acid, followed by centrifugation at 1000× *g* for 10 min. Then, 250 µL of the supernatant was mixed with 250 µL of distilled water and 100 µL of 0.1% FeCl_3_. Finally, 200 µL of this mixture was transferred to a 96-well plate and the absorbance was measured at 700 nm. All values were expressed as milligrams of Trolox equivalents per gram of oil (mg Trolox/g oil).

### 4.4. In Vivo Study

#### 4.4.1. Animal Model

Male C57BL/6J mice aged between 12 and 16 weeks, weighing approximately 25–44 g, were acquired from Pasteur Medical Laboratory in Casablanca, Morocco. The experimental procedure was performed following the instructions of the Institutional Animal Ethics Committee of Moroccan Association For Research and Ethics (Animal Ethics code: 25 May 2023 11-REC-23/Animal/PPL/70/8647). All animals were healthy, immunocompetent, and free of known pathogens at the time of arrival. The mice were not genetically modified and had not been subjected to any prior experimental procedures before the start of this study. All animals were housed in a 12 h light/12 h dark cycle at a temperature of 22 ± 2 °C, a relative humidity of 45–65%, and were fed a standard diet and water ad libitum.

#### 4.4.2. Experimental Design

Three weeks after acclimatization, 59 male C57BL/6J mice were manually assigned to ten experimental groups (4–6 mice/group). The animals were housed in numbered cages according to the treatment administered. Group allocation was known to the researcher during the allocation and conduct of the experiment. However, individuals responsible for outcome assessment and data analysis were blinded to group assignments to minimize potential bias.

**Figure 10:**
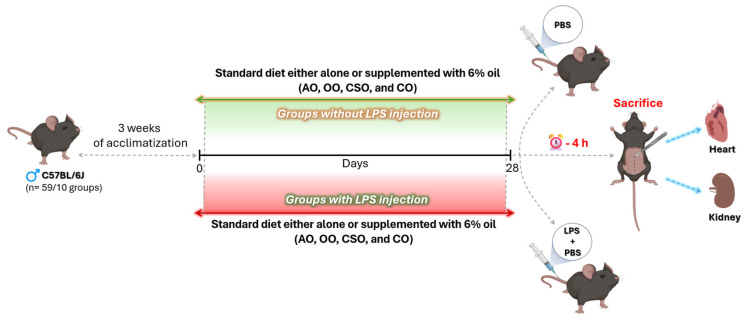
In vivo experimental design schematic illustrating the grouping and treatment protocol of male C57BL/6J mice. Following a 3-week acclimatization, animals were randomly assigned to ten experimental groups (4–6 mice) and received the following for 28 days: a standard chow (2 groups, control); a standard chow supplemented with 6% (*w*/*w*) argan oil (2 groups, AO); a standard chow supplemented with 6% (*w*/*w*) olive oil (2 groups, OO); a standard chow supplemented with 6% (*w/w*) cactus seed oil (2 groups, CSO); or a standard chow supplemented with 6% (*w*/*w*) colza oil (2 groups, CO). Oils were solubilized in acetone at a 1:4 (*v*/*v*) ratio prior to incorporation into the feed. On day 28, four hours before euthanasia and during the fed state, one group from the control (+LPS), AO (AO+LPS), OO (OO+LPS), CSO (CSO+LPS) and CO (CO+LPS) groups received intraperitoneal injections (5 mg/kg) of 100 μg of *Escherichia coli* 0111:B4 LPS (Sigma, Saint-Quentin-Fallavier, France) resuspended in PBS or an equal volume of PBS alone. After sacrifice, heart and kidney tissues were immediately frozen in an ethanol–dry ice bath and stored at −80 °C for biochemical analyses.

Each group received a standard chow diet (PAM 72, Alf Sahel, Morocco) for 28 days (Figure 10), either alone or supplemented with 6% (*w*/*w*) of one of four vegetable oils: AO, OO, CSO, and CO. The oils were solubilized in acetone at a 1:4 (*v*/*v*) ratio prior to incorporation into the feed. This mixture was added to diet pellets and then evaporated overnight. The 6% (*w*/*w*) oil concentration corresponds to 6 g of oil per 95 g of diet, approximately 6.53 mL of oil, as described by El Kebbaj et al. [46]. The experimental groups included two control groups receiving standard chow without oil supplementation, and pairs of groups for each oil type (AO, OO, CSO, CO) as follows: 2 control groups fed with a standard diet; 2 argan oil groups fed with a standard diet supplemented with 6% (*w*/*w*) AO; 2 olive oil groups fed with a standard diet supplemented with 6% (*w*/*w*) OO; 2 cactus seed oil groups fed with a standard diet supplemented with 6% (*w*/*w*) CSO; and 2 colza oil groups fed with a standard diet supplemented with 6% (*w*/*w*) CO.

Four hours before euthanasia and in the fed state, one group from each of the treatment groups (control (+LPS), AO (AO+LPS), OO (OO+LPS), CSO (CSO+LPS), and CO (CO+LPS)) received an injection (5 mg/kg) via the tail vein of 100 μg of LPS from *Escherichia coli* O111:B4 (Sigma, Saint-Quentin-Fallavier, France) prepared in sterile phosphate-buffered saline (PBS). Negative control groups received an equal volume of PBS alone. The LPS serotype of *Escherichia coli* O111:B4 has already been used in the treatment of mice according to other studies and our previous studies [9,10,11,46,110,111]. After euthanasia, the hearts and kidneys of mice were collected, frozen in an ethanol–dry ice bath and stored at −80 °C.

#### 4.4.3. Preparation of Tissue Homogenates

Homogenates were prepared from cardiac and renal tissues using a Potter–Elvehjem homogenizer (Dominique Dutscher, Issy-les-Moulineaux, France) equipped with a PTFE pestle and a borosilicate glass mortar, with a piston–cylinder clearance of 0.1–0.15 mm to ensure optimal cellular homogenization. Tissues were diluted in phosphate buffer (KH_2_PO_4_/K_2_HPO_4_, 50 mM, pH 7.4) to obtain a 10% (*w*/*v*) dilution for heart and 20% (*w*/*v*) for kidneys, allowing plasma membrane disruption and release of cellular contents. The resulting homogenates were subsequently sonicated using an OMNI Sonic Ruptor 4000 (Omni International, Kennesaw, GA, USA) equipped with a probe, set at 100 W (20 kHz), for three 10-s cycles with cooling on ice between cycles to prevent overheating and ensure complete cell lysis. Finally, the samples were centrifuged at 3000× *g* for 10 min at 4 °C. The resulting supernatants were collected as tissue extracts and immediately frozen until further analysis.

#### 4.4.4. Quantification of Protein

Protein content was quantified using the PierceTM BCA Protein Assay Kit (Ref. 23227, LOT XB338073) (ThermoFisher Scientific, Illkirch, France).

#### 4.4.5. Measurement of Enzymatic Antioxidants Defense

##### Superoxide Dismutase (SOD) Activity

SOD activity was assayed following the method of Beyer and Fridovich [112], based on the inhibition of nitro blue tetrazolium (NBT) reduction to formazan blue by superoxide anions (O_2_^•−^) generated through a light-induced reaction using L-methionine and riboflavin. The reaction mixture included phosphate buffer (116 mM, pH 7.4), EDTA (0.1 mM), L-methionine (12 mM), nitro blue tetrazolium (75 mM), Triton X-100 (0.025%), riboflavin (2 µM), and tissue homogenate. After 10 min of light exposure, absorbance was measured at 560 nm (infinite M200 PRO, TECAN, Zürich, Switzerland). SOD activity was expressed as the amount of enzyme required to inhibit 50% of NBT reduction, in units per milligram of protein (U/mg protein).

##### Catalase (CAT) Activity

CAT activity was determined by monitoring the decomposition of H_2_O_2_ through the decrease in absorbance at 240 nm, as described by Beers and Sizer [113]. The reaction was performed in a UV-transparent 96-well plate (Greiner Bio-One, Kremsmünster, Austria, Ref. 655801), with 170 µL of Tris-HCl buffer (1 M, pH 7.4), 10 µL of H_2_O_2_ (400 mM), and 10 µL of protein extract in a final volume of 200 µL. Absorbance was recorded at 240 nm for 2 min at 37 °C using a plate reader (infinite M200 PRO, TECAN, Zürich, Switzerland). Catalase specific activity was expressed as nanomoles of H_2_O_2_ degraded per minute per milligram of protein (U/mg protein).

##### Glutathione Peroxidase (GPx) Activity

GPx activity was measured as described by Mills [114], based on the reduction of H_2_O_2_ by GPx using GSH as a cofactor. The remaining GSH reacts with 5,5′-dithiobis-(2-nitrobenzoic acid) (DTNB), forming a yellow chromophore measured at 412 nm. The reaction mixture (15 µL homogenate, 15 µL phosphate buffer 0.4 M, pH 7.4, 10 µL GSH 30 mM, 5 µL sodium azide 10 mM, and 5 µL H_2_O_2_ 1 mM) was incubated at 37 °C for 15 min. After stopping the reaction with 25 µL of 5% TCA and centrifuging at 3000× *g* for 5 min, 20 µL of supernatant was mixed with 40 µL of phosphate buffer (50 mM, pH 7.4) and 140 µL of DTNB (0.04%) in a 96-well plate. Absorbance was read at 412 nm (infinite M200 PRO, TECAN, Zürich, Switzerland), and GPx activity was expressed as micromoles of GSH per minute per milligram of protein (U/mg protein).

#### 4.4.6. Measurement of Reduced Glutathione (GSH)

GSH levels were determined as described by Ellman [115], based on the reaction between GSH and DTNB, forming a yellow-colored 5-thiol-2-nitrobenzoate anion (TNB^−^) product measured at 412 nm (infinite M200 PRO, TECAN, Zürich, Switzerland). Briefly, 60 µL of homogenate was mixed with 30 µL of 5% trichloroacetic acid, centrifuged (10 min at 12,000× *g* at 4 °C), and 10 µL of the supernatant was mixed with 170 µL of phosphate buffer (50 mM, pH = 7.4) and 20 µL of DTNB (6 mM). Absorbance was read at 412 nm, and GSH concentration was expressed as micromoles of GSH per milligram of protein (µmol GSH/mg protein).

#### 4.4.7. Measurement of Monodialdehyde (MDA)

MDA levels, a biomarker of lipid peroxidation, were estimated using the Ohkawa method [116]. 75 µL of homogenate were mixed with 75 µL of 5% TCA and 150 µL of 0.67% TBA, heated at 100 °C for 15 min, then rapidly cooled. After adding 600 µL of n-butanol, samples were centrifuged (3000× *g*, 15 min), and the upper organic phase was collected for absorbance reading at 532 nm (infinite M200 PRO, TECAN, Zürich, Switzerland). MDA was expressed as nanomoles of MDA per milligram of protein (nmol MDA/mg protein).

### 4.5. Statistical Analysis

All data are presented as mean ± standard deviation (SD). Statistical analyses were performed using GraphPad Prism software (version 8.0; San Diego, CA, USA). The differences between experimental groups were assessed by two-way analysis of variance (ANOVA) followed by Tukey’s multiple comparisons test. A *p*-value ≤ 0.05 was considered statistically significant. Additionally, the heatmaps, Pearson correlation analysis, and PCA (Principal Component Analysis) were conducted using R software (version 4.4.1). Raw biochemical data (SOD, catalase, GPx, GSH, and MDA levels) obtained from heart and kidney tissues under different treatments were first normalized using z-score transformation to standardize the scale across parameters. The heatmap was generated using the “pheatmap” and “ggplot2” packages, with color gradients (red to blue) representing the relative increase or decrease of each parameter compared to the overall mean. Hierarchical clustering was performed on both rows (treatment groups) and columns (parameters) using the Ward.D2 linkage method and Euclidean distance. The Pearson correlation analysis was visualized through heatmaps generated with the “corrplot” package, where the color gradient (red to blue) reflects the strength and direction of correlation coefficients.

## 5. Conclusions

In summary, our findings provide a comprehensive comparative assessment of the antioxidant potential of AO, OO, CSO, and CO evaluated both in vitro and in vivo, with particular attention to their total oils and respective lipophilic and methanolic fractions. AO exhibited a unique fatty acid profile, with a balanced distribution of oleic acid (43.52%) and linoleic acid (35.39%), and a relevant level of γ-tocopherol. These compositional features were reflected in its strong antioxidant performance across DPPH, ABTS, and FRAP assays, especially in the methanolic fraction, which was consistently richer in polyphenols. Importantly, our in vivo study demonstrates that acute LPS exposure induces significant oxidative stress in both murine heart and kidney tissues, as evidenced by a pronounced increase in enzymatic antioxidants—SOD, CAT, and GPx activities—as well as elevated lipid peroxidation (MDA levels). Notably, the kidney exhibited a more robust enzymatic response compared to the heart, while GSH levels showed tissue-specific variations, decreasing significantly in the heart but increasing in the kidney, likely reflecting distinct adaptive mechanisms. Administration of the oils alone under physiological conditions did not significantly alter the antioxidant enzyme activities or oxidative stress markers, indicating a lack of basal modulation in healthy tissue. However, co-treatment of LPS-challenged mice with these oils markedly attenuated the LPS-induced oxidative perturbations. Among the vegetable oils tested, AO consistently demonstrated superior protective effects. AO co-treatment notably restored SOD, CAT, and GPx activities closer to control levels, reduced lipid peroxidation substantially, and showed a trend towards normalizing GSH depletion in the heart. In comparison, OO, CSO, and CO also mitigated oxidative stress but with less pronounced effects and some tissue-specific variability. Multivariate analyses highlighted strong positive correlations among enzymatic antioxidants and MDA, indicating a coordinated response to oxidative stress. PCA revealed distinct clustering patterns, with AO co-treatment samples clustering nearest to control groups, emphasizing its potent antioxidant capacity in restoring redox balance.

This study is the first to provide a direct comparison of AO with OO, CSO, and CO regarding their capacity to modulate key oxidative stress parameters following an acute inflammatory insult. It also underscores the importance of evaluating both enzymatic (SOD, CAT, GPx) and non-enzymatic (GSH) antioxidants, along with lipid peroxidation to comprehensively assess tissue-specific responses. Taken together, our results strongly support the antioxidant and protective potential of AO against acute LPS-induced oxidative damage in vital organs, suggesting its promising role as a dietary agent to prevent or modulate oxidative stress-related conditions.

Although this study was conducted in a murine model and does not explore specific molecular mechanisms or clinical relevance, it lays the groundwork for future investigations. These should aim to identify the signaling pathways involved and assess the translational potential of these oils in clinical or nutritional contexts. Taken together, our results provide scientific evidence supporting the antioxidant and protective potential of these natural oils, particularly in the context of acute oxidative stress. This study contributes to the scientific valorization of natural oils such as argan oil and supports their consideration as promising dietary components in the prevention or modulation of oxidative stress-related conditions.

## Figures and Tables

**Table 1:** Composition (%) of AO (argan oil), OO (olive oil), CSO (cactus seed oil), and CO (colza oil).

	AO	OO	CSO [61]	CO [61]
**Fatty acids (%)**				
Myristic acid C14:0	0.18	nd	0.14	0.12
Pentadecanoic acid C15:0	0.06	nd	nd	nd
Palmitic acid C16:0	12.99	6.02	13.85	6.48
Stearic acid C18:0	6.71	nd	4.61	1.57
Oleic acid C18:1, ∆9	43.52	62.31	9.95	77.18
Linoleic acid C18:2, ∆6	35.39	0.69	67.32	nd
Arachidic acid C20:0	0.55	nd	0.58	2.38
Behenic acid C22:0	0.14	nd	0.28	1.32
Lignoceric acid C24:0	0.05	nd	0.26	0.46
**Phytosterol (%)**				
Stigmast-7-en-3-ol	0.05	nd	nd	nd
**Tocopherol (%)**				
γ-Tocopherol	0.02	nd	0.08	0.15
**Triterpene (%)**				
Squalene	0.33	28.27	nd	nd

nd: not detected.

**Table 2:** Total phenolic content (TPC) and antioxidant activities (ABTS, DPPH, FRAP) of all fractions (mean ± SD, n = 3).

		TPC (mg GAE/Kg Oil)	ABTS (% Inhibition)	DPPH (% Inhibition)	FRAP (mg Trolox/g Oil)
**MF**	**AO**	80 ± 5.6	74.4 ± 0.61	57.6 ± 2.73	0.46 ± 0.06
	**OO**	94.3 ± 4.9	91 ± 0.41	69.1± 3.51	1.56 ± 0.15
	**CSO**	59.3 ± 1.8	60.8 ± 1.16	71.9 ± 1.2	0.44 ± 0.18
	**CO**	54.7 ± 0.8	65 ± 0.73	58 ± 2.15	0.37 ± 0.03
**LF**	**AO**	57.7 ± 3.5	58.7 ± 0.8	34.8 ± 3.94	0.49 ± 0.04
	**OO**	68.87 ± 0.6	62.4 ± 0.57	62.1 ± 4.46	0.67 ± 0.05
	**CSO**	55.8 ± 1.3	46.4 ± 2.26	15.7 ± 3.46	0.41 ± 0.04
	**CO**	45.3 ± 0.6	51.57 ± 0.96	44.28 ± 4	0.41 ± 0.03
**TF**	**AO**	73.1 ± 1.8	64 ± 3.75	55.4 ± 7.6	0.41 ± 0.05
	**OO**	82.1 ± 4.1	66.17 ± 2.13	66.4 ± 1.94	1.15 ± 0.1
	**CSO**	44 ± 0.15	60.2 ± 1.24	34.9 ± 0.9	0.50 ± 0.06
	**CO**	48.7 ± 0.8	60.1 ± 0.81	54.2 ± 1.91	0.55 ± 0.05

TPC: Total phenolic content; MF: Methanolic fraction; LF: Lipidic fraction; TF: Total fraction; AO: Argan oil; OO: Olive oil; CSO: Cactus seed oil; CO: Colza oil.

**Table 3:** Acidity, peroxide value, and specific extinction coefficient of AO, OO, CSO, and CO.

	AO [44]	OO [44]	CSO [44]	CO [103]
**Acidity (%, as oleic acid)**	0.28 ± 0.00	1.05 ± 0.00	0.77 ± 0.00	1.41 ± 0.10
**Peroxide value (meq O_2_/kg oil)**	2.42 ± 0.04	2.26 ± 0.05	2.84 ± 0.05	3.7 ± 0.39
**K232**	1.04 ± 0.09	1.21 ± 0.05	1.83 ± 0.06	2.555 ± 0.04
**K270**	0.19 ± 0.00	0.14 ± 0.00	0.23 ± 0.00	0.544 ± 0.021

AO: Argan oil; OO: Olive oil; CSO: Cactus seed oil; CO: Colza oil.

## Data Availability

Data are contained within the article.

## Supplementary Materials

The following supporting information can be downloaded at: https://www.mdpi.com/article/10.3390/ijms26178300/s1.

## Abbreviations

The following abbreviations are used in this manuscript:

AO	Argan oil
OO	Olive oil
CSO	Cactus seed oil
CO	Colza oil
LPS	Lipopolysaccharide
MF	Methanolic fraction
LF	Lipidic fraction
TF	Total fraction
TPC	Total phenolic content
ROS	Reactive oxygen species
SOD	Superoxide dismutase
CAT	Catalase
GPx	Glutathione peroxidase
GSH	Reduced glutathione
MDA	Malondialdehyde
Nrf2	Nuclear factor erythroid 2-related factor 2
FOXO	Forkhead box O
HDL	High-density lipoprotein
LDL	Low-density lipoprotein
O_2_^•−^	Superoxide anion
H_2_O_2_	Hydrogen peroxide
^•^OH	Hydroxyl radical
ABTS	2,2′-azinobis(3 ethylbenzothiazoline-6-sulfonic acid) diammonium
DPPH	2,2-diphenyl-1-picrylhydrazyl radical
FRAP	Ferric reducing antioxidant power
TBA	Thiobarbituric acid
TCA	Trichloroacetic acid
DTNB	5,5′-dithiobis(2-nitrobenzoic acid)
TNB^−^	5-thio-2-nitrobenzoate
BCA	Bicinchoninic acid
ANOVA	Analysis of variance
SD	Standard deviation
PCA	Principal component analysis

## References

[1] Heurtaux T., Bouvier D.S., Benani A., Helgueta Romero S., Frauenknecht K.B.M., Mittelbronn M., Sinkkonen L. (2022). Normal and Pathological NRF2 Signalling in the Central Nervous System. Antioxidants.

[2] Juan C.A., Pérez de la Lastra J.M., Plou F.J., Pérez-Lebeña E. (2021). The Chemistry of Reactive Oxygen Species (ROS) Revisited: Outlining Their Role in Biological Macromolecules (DNA, Lipids and Proteins) and Induced Pathologies. Int. J. Mol. Sci..

[3] Burton G.J., Jauniaux E. (2011). Oxidative Stress. Best Pract. Res. Clin. Obstet. Gynaecol..

[4] Jiménez-Fernández S., Gurpegui M., Garrote-Rojas D., Gutiérrez-Rojas L., Carretero M.D., Correll C.U. (2021). Oxidative Stress Parameters and Antioxidants in Patients with Bipolar Disorder: Results from a Meta-Analysis Comparing Patients, Including Stratification by Polarity and Euthymic Status, with Healthy Controls. Bipolar Disord..

[5] Liu T., Sun L., Zhang Y., Wang Y., Zheng J. (2022). Imbalanced GSH/ROS and Sequential Cell Death. J. Biochem. Mol. Toxicol..

[6] Fransen M., Lismont C. (2018). Peroxisomes and Cellular Oxidant/Antioxidant Balance: Protein Redox Modifications and Impact on Inter-Organelle Communication. Subcell. Biochem..

[7] Vamecq J., Andreoletti P., El Kebbaj R., Saih F.-E., Latruffe N., El Kebbaj M.H.S., Lizard G., Nasser B., Cherkaoui-Malki M. (2018). Peroxisomal Acyl-CoA Oxidase Type 1: Anti-Inflammatory and Anti-Aging Properties with a Special Emphasis on Studies with LPS and Argan Oil as a Model Transposable to Aging. Oxid. Med. Cell. Longev..

[8] Schrader M., Fahimi H.D. (2006). Peroxisomes and Oxidative Stress. Biochim. Biophys. Acta.

[9] El Kamouni S., El Kebbaj R., Andreoletti P., El Ktaibi A., Rharrassi I., Essamadi A., El Kebbaj M.S., Mandard S., Latruffe N., Vamecq J. (2017). Protective Effect of Argan and Olive Oils against LPS-Induced Oxidative Stress and Inflammation in Mice Livers. Int. J. Mol. Sci..

[10] Tahri-Joutey M., Saih F.-E., El Kebbaj R., Gondcaille C., Vamecq J., Latruffe N., Lizard G., Savary S., Nasser B., Cherkaoui-Malki M. (2022). Protective Effect of Nopal Cactus (Opuntia Ficus-Indica) Seed Oil against Short-Term Lipopolysaccharides-Induced Inflammation and Peroxisomal Functions Dysregulation in Mouse Brain and Liver. Int. J. Mol. Sci..

[11] Essadek S., Bouchab H., El Kebbaj R., Gondcaille C., El Kamouni S., Savary S., Vamecq J., Essamadi A., Cherkaoui-Malki M., Nasser B. (2022). Effects of a Short-Term Lipopolysaccharides Challenge on Mouse Brain and Liver Peroxisomal Antioxidant and β-oxidative Functions: Protective Action of Argan Oil. Pharmaceuticals.

[12] Basauri A., González-Fernández C., Fallanza M., Bringas E., Fernandez-Lopez R., Giner L., Moncalián G., De La Cruz F., Ortiz I. (2020). Biochemical Interactions between LPS and LPS-Binding Molecules. Crit. Rev. Biotechnol..

[13] Ciesielska A., Matyjek M., Kwiatkowska K. (2021). TLR4 and CD14 Trafficking and Its Influence on LPS-Induced pro-Inflammatory Signaling. Cell. Mol. Life Sci..

[14] Atrooz F., Salim S. (2020). Sleep Deprivation, Oxidative Stress and Inflammation. Adv. Protein Chem. Struct. Biol..

[15] Paris-Robidas S., Bolduc I., Lapointe V., Galimi J., Lemieux P., Huppé C.-A., Couture F. (2024). Impact of Time Intervals on Drug Efficacy and Phenotypic Outcomes in Acute Respiratory Distress Syndrome in Mice. Sci. Rep..

[16] Steven S., Dib M., Roohani S., Kashani F., Münzel T., Daiber A. (2017). Time Response of Oxidative/Nitrosative Stress and Inflammation in LPS-Induced Endotoxaemia—A Comparative Study of Mice and Rats. Int. J. Mol. Sci..

[17] Seese M.H., Steelman A.J., Erdman J.W. (2024). The Impact of LPS on Inflammatory Responses in Alpha-Tocopherol Deficient Mice. Curr. Dev. Nutr..

[18] Suliman H.B., Welty-Wolf K.E., Carraway M., Tatro L., Piantadosi C.A. (2004). Lipopolysaccharide Induces Oxidative Cardiac Mitochondrial Damage and Biogenesis. Cardiovasc. Res..

[19] Asgharzadeh F., Bargi R., Hosseini M., Farzadnia M., Khazaei M. (2018). Cardiac and Renal Fibrosis and Oxidative Stress Balance in Lipopolysaccharide-Induced Inflammation in Male Rats. ARYA Atheroscler..

[20] Ben-Shaul V., Lomnitski L., Nyska A., Zurovsky Y., Bergman M., Grossman S. (2001). The Effect of Natural Antioxidants, NAO and Apocynin, on Oxidative Stress in the Rat Heart Following LPS Challenge. Toxicol. Lett..

[21] Khodir A., Ghoneim H., Rahim M., Suddek G. (2016). Montelukast Attenuates Lipopolysaccharide-Induced Cardiac Injury in Rats. Hum. Exp. Toxicol..

[22] Feingold K.R., Wang Y., Moser A., Shigenaga J.K., Grunfeld C. (2008). LPS Decreases Fatty Acid Oxidation and Nuclear Hormone Receptors in the Kidney. J. Lipid Res..

[23] Sriskandan S., Altmann D.M. (2008). The Immunology of Sepsis. J. Pathol..

[24] Rabbaa S., Bouchab H., Laaziouez Y., Limami Y., Nasser B., Andreoletti P., Cherkaoui-Malki M., El Kebbaj R. (2025). Argan Oil: A Natural Bioactive Lipid Modulating Oxidative Stress and Inflammation. Antioxidants.

[25] Arulselvan P., Fard M.T., Tan W.S., Gothai S., Fakurazi S., Norhaizan M.E., Kumar S.S. (2016). Role of Antioxidants and Natural Products in Inflammation. Oxid. Med. Cell. Longev..

[26] Lobo V., Patil A., Phatak A., Chandra N. (2010). Free Radicals, Antioxidants and Functional Foods: Impact on Human Health. Pharmacogn. Rev..

[27] Nesci S., Spagnoletta A., Oppedisano F. (2023). Inflammation, Mitochondria and Natural Compounds Together in the Circle of Trust. Int. J. Mol. Sci..

[28] Sheikh N.A., Desai T.R., Tirgar P.R. (2017). Evaluation of Iron Chelating and Antioxidant Potential of Epilobium Hirsutum for the Management of Iron Overload Disease. Biomed. Pharmacother..

[29] Tabolacci C., Forni C., Jadeja R.N., Facchiano F. (2019). Natural Compounds against Cancer, Inflammation, and Oxidative Stress. BioMed Res. Int..

[30] Minasyan A., Pires V., Gondcaille C., Ginovyan M., Mróz M., Savary S., Cherkaoui-Malki M., Kusznierewicz B., Bartoszek A., Andreoletti P. (2025). Ribes Nigrum Leaf Extract Downregulates Pro-Inflammatory Gene Expression and Regulates Redox Balance in Microglial Cells. BMC Complement. Med. Ther..

[31] El Kebbaj R., Bouchab H., Tahri-Joutey M., Rabbaa S., Limami Y., Nasser B., Egbujor M.C., Tucci P., Andreoletti P., Saso L. (2024). The Potential Role of Major Argan Oil Compounds as Nrf2 Regulators and Their Antioxidant Effects. Antioxidants.

[32] Gabriele M., Pucci L. (2017). Diet Bioactive Compounds: Implications for Oxidative Stress and Inflammation in the Vascular System. Endocr. Metab. Immune Disord. Drug Targets.

[33] Mattioli R., Mosca L., Sánchez-Lamar A., Tempera I., Hausmann R. (2018). Natural Bioactive Compounds Acting against Oxidative Stress in Chronic, Degenerative, and Infectious Diseases. Oxid. Med. Cell. Longev..

[34] Liang B., Zhu Y.-C., Lu J., Gu N. (2021). Effects of Traditional Chinese Medication-Based Bioactive Compounds on Cellular and Molecular Mechanisms of Oxidative Stress. Oxid. Med. Cell. Longev..

[35] Benayad S., Es-Sai B., Laaziouez Y., Rabbaa S., Wahnou H., Bouchab H., El Attar H., Benabdelkhalek B., Amahdar L., Abboussi O. (2025). Protective Effects of Sodium Copper Chlorophyllin and/or Ascorbic Acid Against Barium Chloride-Induced Oxidative Stress in Mouse Brain and Liver. Molecules.

[36] El Ghachi H., Oukhrib M., Aziz F., Benrazzouk K., Gamrani H., Soulimani R., Boukhzar L. (2025). Exploring the Phytochemical and Toxicological Profile of Moroccan Cannabis Sativa L. Leaves Extract: Behavioral, Histological, and Oxidative Stress Assessments. J. Ethnopharmacol..

[37] Khan M.Z., Khan A., Huang B., Wei R., Kou X., Wang X., Chen W., Li L., Zahoor M., Wang C. (2024). Bioactive Compounds Protect Mammalian Reproductive Cells from Xenobiotics and Heat Stress-Induced Oxidative Distress via Nrf2 Signaling Activation: A Narrative Review. Antioxidants.

[38] El Kebbaj R., Kamouni S.E., Hajj H.I.E., Andreoletti P., Gresti J., Latruffe N., El Kebbaj M.S., Vamecq J., Lizard G., Nasser B. (2013). Modulation of Peroxisomes Abundance by Argan Oil and Lipopolysaccharides in Acyl-CoA Oxidase 1-Deficient Fibroblasts. Health.

[39] Kadda S., Belabed A., Loukili E.H., Hammouti B., Fadlaoui S. (2022). Temperature and Extraction Methods Effects on Yields, Fatty Acids, and Tocopherols of Prickly Pear (*Opuntia Ficus-Indica* L.) Seed Oil of Eastern Region of Morocco. Environ. Sci. Pollut. Res. Int..

[40] Al-Naqeb G., Fiori L., Ciolli M., Aprea E. (2021). Prickly Pear Seed Oil Extraction, Chemical Characterization and Potential Health Benefits. Molecules.

[41] Mechqoq H., El Yaagoubi M., El Hamdaoui A., Momchilova S., da Guedes Silva Almeida J.R., Msanda F., El Aouad N. (2021). Ethnobotany, Phytochemistry and Biological Properties of Argan Tree (*Argania spinosa* (L.) Skeels) (Sapotaceae)—A Review. J. Ethnopharmacol..

[42] Charrouf Z., Guillaume D. (2010). Should the Amazigh Diet (Regular and Moderate Argan-Oil Consumption) Have a Beneficial Impact on Human Health?. Crit. Rev. Food Sci. Nutr..

[43] Makbal R., Idrissi F.E.J., Ouchbani T., Tastift M.A., Kiai H., Hafidi A., Gadhi C. (2021). Anti-Inflammatory, Antioxidant, Chemical Characterization, and Safety Assessment of *Argania spinosa* Fruit Shell Extract from South-Western Morocco. BioMed Res. Int..

[44] El Kharrassi Y., Maata N., Mazri M.A., El Kamouni S., Talbi M., El Kebbaj R., Moustaid K., Essamadi A.K., Andreoletti P., El Mzouri E.H. (2018). Chemical and Phytochemical Characterizations of Argan Oil (*Argania spinosa* L. Skeels), Olive Oil (*Olea europaea* L. Cv. Moroccan picholine), Cactus Pear (Opuntia megacantha salm-dyck) Seed Oil and Cactus Cladode Essential Oil. J. Food Meas. Charact..

[45] Mouas N.T., Kabouche Z., Bellel N., Chertout L.K. Opuntia Ficus-Indica a Mediterranean Diet Product. Proceedings of the 1st International Electronic Conference on Biological Diversity, Ecology and Evolution.

[46] El Kebbaj R., Andreoletti P., El Hajj H.I., El Kharrassi Y., Vamecq J., Mandard S., Saih F.-E., Latruffe N., El Kebbaj M.S., Lizard G. (2015). Argan Oil Prevents Down-Regulation Induced by Endotoxin on Liver Fatty Acid Oxidation and Gluconeogenesis and on Peroxisome Proliferator-Activated Receptor Gamma Coactivator-1α, (PGC-1α), Peroxisome Proliferator-Activated Receptor α (PPARα) and Estrogen Related Receptor α (ERRα). Biochim. Open.

[47] Derouiche A., Cherki M., Drissi A., Bamou Y., El Messal M., Idrissi-Oudghiri A., Lecerf J.M., Adlouni A. (2005). Nutritional Intervention Study with Argan Oil in Man: Effects on Lipids and Apolipoproteins. Ann. Nutr. Metab..

[48] Ursoniu S., Sahebkar A., Serban M.-C., Banach M., Lipid and Blood Pressure Meta-analysis Collaboration Group (2018). The Impact of Argan Oil on Plasma Lipids in Humans: Systematic Review and Meta-Analysis of Randomized Controlled Trials: Lipid-Modifying Effects of Argan Oil. Phytother. Res..

[49] Ould Mohamedou M.M., Zouirech K., El Messal M., El Kebbaj M.S., Chraibi A., Adlouni A. (2011). Argan Oil Exerts an Antiatherogenic Effect by Improving Lipids and Susceptibility of LDL to Oxidation in Type 2 Diabetes Patients. Int. J. Endocrinol..

[50] Podadera-Herreros A., Alcala-Diaz J.F., Gutierrez-Mariscal F.M., Jimenez-Torres J., de la Cruz-Ares S., Larriva A.P.A., Cardelo M.P., Torres-Peña J.D., Luque R.M., Ordovas J.M. (2022). Long-Term Consumption of a Mediterranean Diet or a Low-Fat Diet on Kidney Function in Coronary Heart Disease Patients: The CORDIOPREV Randomized Controlled Trial. Clin. Nutr..

[51] Doménech M., Roman P., Lapetra J., García de la Corte F.J., Sala-Vila A., de la Torre R., Corella D., Salas-Salvadó J., Ruiz-Gutiérrez V., Lamuela-Raventós R.-M. (2014). Mediterranean Diet Reduces 24-Hour Ambulatory Blood Pressure, Blood Glucose, and Lipids: One-Year Randomized, Clinical Trial. Hypertens. Dallas Tex 1979.

[52] Estruch R., Ros E., Salas-Salvadó J., Covas M.-I., Corella D., Arós F., Gómez-Gracia E., Ruiz-Gutiérrez V., Fiol M., Lapetra J. (2018). Primary Prevention of Cardiovascular Disease with a Mediterranean Diet Supplemented with Extra-Virgin Olive Oil or Nuts. N. Engl. J. Med..

[53] Covas M.-I. (2008). Bioactive Effects of Olive Oil Phenolic Compounds in Humans: Reduction of Heart Disease Factors and Oxidative Damage. Inflammopharmacology.

[54] Schwingshackl L., Morze J., Hoffmann G. (2020). Mediterranean Diet and Health Status: Active Ingredients and Pharmacological Mechanisms. Br. J. Pharmacol..

[55] Schwingshackl L., Krause M., Schmucker C., Hoffmann G., Rücker G., Meerpohl J.J. (2019). Impact of Different Types of Olive Oil on Cardiovascular Risk Factors: A Systematic Review and Network Meta-Analysis. Nutr. Metab. Cardiovasc. Dis..

[56] Amiri M., Raeisi-Dehkordi H., Moghtaderi F., Zimorovat A., Mohyadini M., Salehi-Abargouei A. (2022). The Effects of Sesame, Canola, and Sesame–Canola Oils on Cardiometabolic Markers in Patients with Type 2 Diabetes: A Tri-ple-Blind Three-Way Randomized Crossover Clinical Trial. Eur. J. Nutr..

[57] Nicol K., Mansoorian B., Latosinska A., Koutroulaki A., Mullen B., Combet E. (2022). No Evidence of Differential Impact of Sunflower and Rapeseed Oil on Biomarkers of Coronary Artery Disease or Chronic Kidney Disease in Healthy Adults with Overweight and Obesity: Result from a Randomised Control Trial. Eur. J. Nutr..

[58] Nielsen N.S., Pedersen A., Sandström B., Marckmann P., Høy C.-E. (2002). Different Effects of Diets Rich in Olive Oil, Rapeseed Oil and Sunflower-Seed Oil on Postprandial Lipid and Lipoprotein Concentrations and on Lipoprotein Oxidation Susceptibility. Br. J. Nutr..

[59] Bakour M., Al-Waili N., El-Haskoury R., El-Menyiy N., Al-Waili T., AL-Waili A., Lyoussi B. (2017). Comparison of Hypotensive, Diuretic and Renal Effects between Cladodes of *Opuntia Ficus-Indica* and Furosemide. Asian Pac. J. Trop. Med..

[60] Marrone G., Murri A., Urciuoli S., Di Lauro M., Grazioli E., Vignolini P., Cornali K., Tranchita E., Masci C., Cerulli C. (2024). Functional Foods and Adapted Physical Activity as New Adjuvant Therapy for Chronic Kidney Disease Patients. Nutrients.

[61] Bouchab H., Ishaq A., Limami Y., Saretzki G., Nasser B., El Kebbaj R. (2024). Antioxidant Effects of Cactus Seed Oil against Iron-Induced Oxidative Stress in Mouse Liver, Brain and Kidney. Molecules.

[62] Miao L., St Clair D.K. (2009). Regulation of Superoxide Dismutase Genes: Implications in Disease. Free Radic. Biol. Med..

[63] Glorieux C., Calderon P.B. (2017). Catalase, a Remarkable Enzyme: Targeting the Oldest Antioxidant Enzyme to Find a New Cancer Treatment Approach. Biol. Chem..

[64] Lubos E., Loscalzo J., Handy D.E. (2011). Glutathione Peroxidase-1 in Health and Disease: From Molecular Mechanisms to Therapeutic Opportunities. Antioxid. Redox Signal..

[65] Forman H.J., Zhang H., Rinna A. (2009). Glutathione: Overview of Its Protective Roles, Measurement, and Biosynthesis. Mol. Aspects Med..

[66] Khallouki F., Younos C., Soulimani R., Oster T., Charrouf Z., Spiegelhalder B., Bartsch H., Owen R.W. (2003). Consumption of Argan Oil (Morocco) with Its Unique Profile of Fatty Acids, Tocopherols, Squalene, Sterols and Phenolic Compounds Should Confer Valuable Cancer Chemopreventive Effects. Eur. J. Cancer Prev..

[67] Khallouki F., Eddouks M., Mourad A., Breuer A., Owen R.W. (2017). Ethnobotanic, Ethnopharmacologic Aspects and New Phytochemical Insights into Moroccan Argan Fruits. Int. J. Mol. Sci..

[68] Debbabi M., Zarrouk A., Bezine M., Meddeb W., Nury T., Badreddine A., Karym E.M., Sghaier R., Bretillon L., Guyot S. (2017). Comparison of the Effects of Major Fatty Acids Present in the Mediterranean Diet (Oleic Acid, Docosahexaenoic Acid) and in Hydrogenated Oils (Elaidic Acid) on 7-Ketocholesterol-Induced Oxiapoptophagy in Microglial BV-2 Cells. Chem. Phys. Lipids.

[69] Zeng X., Zhu M., Liu X., Chen X., Yuan Y., Li L., Liu J., Lu Y., Cheng J., Chen Y. (2020). Oleic Acid Ameliorates Palmitic Acid Induced Hepatocellular Lipotoxicity by Inhibition of ER Stress and Pyroptosis. Nutr. Metab..

[70] Wang R., Kern J.T., Goodfriend T.L., Ball D.L., Luesch H. (2009). Activation of the Antioxidant Response Element by Specific Oxidized Metabolites of Linoleic Acid. Prostaglandins Leukot. Essent. Fat. Acids.

[71] Zhang Q., Jiang Y., Qin Y., Liu J., Xie Y., Zhang L., Li K., Wang X., Liu G. (2024). Linoleic Acid Alleviates Lipopolysaccharide Induced Acute Liver Injury via Activation of Nrf2. Physiol. Res..

[72] Duthie G.G., McPhail D.B., Morrice P.C., Arthur J.R., Ong A.S.H., Packer L. (1992). Antioxidant Effectiveness of Tocopherol Isomers. Lipid-Soluble Antioxidants: Biochemistry and Clinical Applications.

[73] Es-Sai B., Wahnou H., Benayad S., Rabbaa S., Laaziouez Y., El Kebbaj R., Limami Y., Duval R.E. (2025). Gamma-Tocopherol: A Comprehensive Review of Its Antioxidant, Anti-Inflammatory, and Anticancer Properties. Molecules.

[74] Kamal R., Kharbach M., Vander Heyden Y., Doukkali Z., Ghchime R., Bouklouze A., Cherrah Y., Alaoui K. (2019). In Vivo Anti-Inflammatory Response and Bioactive Compounds’ Profile of Polyphenolic Extracts from Edible Argan Oil (*Argania spinosa* L.), Obtained by Two Extraction Methods. J. Food Biochem..

[75] Ben Mansour R., Ben Slema H., Falleh H., Tounsi M., Kechebar M.S.A., Ksouri R., Megdiche-Ksouri W. (2018). Phytochemical Characteristics, Antioxidant, and Health Properties of Roasted and Unroasted Algerian Argan (*Argania spinosa*) Oil. J. Food Biochem..

[76] Elbir M., Es-Safi N.E., Amhoud A., Mbarki M. (2015). Characterization of Phenolic Compounds in Olive Stones of Three Moroccan Varieties. Maderas Cienc. Tecnol..

[77] El haouhay N., Samaniego-Sánchez C., Asehraou A., Villalón-Mir M., López-García de la Serrana H. (2015). Microbiological Characterization of Picholine Variety Olives and Analysis of Olive Oil Produced in Traditional Oil Mills in Morocco. CyTA-J. Food.

[78] Miliauskas G., Venskutonis P.R., van Beek T.A. (2004). Screening of Radical Scavenging Activity of Some Medicinal and Aromatic Plant Extracts. Food Chem..

[79] Nenadis N., Mastralexi A., Tsimidou M.Z. (2019). Physicochemical Characteristics and Antioxidant Potential of the Greek PDO and PGI Virgin Olive Oils (VOOs). Eur. J. Lipid Sci. Technol..

[80] Marfil R., Giménez R., Martínez O., Bouzas P.R., Rufián-Henares J.A., Mesías M., Cabrera-Vique C. (2011). Determination of Polyphenols, Tocopherols, and Antioxidant Capacity in Virgin Argan Oil (*Argania spinosa*, Skeels). Eur. J. Lipid Sci. Technol..

[81] Houshia O.J., Qutit A., Zaid O., Shqair H., Zaid M. (2014). Determination of Total Polyphenolic Antioxidants Contents in West-Bank Olive Oil. J. Nat. Sci. Res..

[82] Zarrouk A., Martine L., Grégoire S., Nury T., Meddeb W., Camus E., Badreddine A., Durand P., Namsi A., Yammine A. (2019). Profile of Fatty Acids, Tocopherols, Phytosterols and Polyphenols in Mediterranean Oils (Argan Oils, Olive Oils, Milk Thistle Seed Oils and Nigella Seed Oil) and Evaluation of their Antioxidant and Cytoprotective Activities. Curr. Pharm. Des..

[83] Bouchab H., Essadek S., El Kamouni S., Moustaid K., Essamadi A., Andreoletti P., Cherkaoui-Malki M., El Kebbaj R., Nasser B. (2023). Antioxidant Effects of Argan Oil and Olive Oil against Iron-Induced Oxidative Stress: In Vivo and In Vitro Approaches. Molecules.

[84] Widomska J., Gruszecki W.I., Subczynski W.K. (2021). Factors Differentiating the Antioxidant Activity of Macular Xanthophylls in the Human Eye Retina. Antioxidants.

[85] Stahl W., Sies H. (2003). Antioxidant Activity of Carotenoids. Mol. Asp. Med..

[86] Koubaa M., Mhemdi H., Barba F.J., Angelotti A., Bouaziz F., Chaabouni S.E., Vorobiev E. (2017). Seed Oil Extraction from Red Prickly Pear Using Hexane and Supercritical CO_2_: Assessment of Phenolic Compound Composition, Antioxidant and Antibacterial Activities. J. Sci. Food Agric..

[87] Chaalal M., Touati N., Louaileche H. (2012). Extraction of Phenolic Compounds and in Vitro Antioxidant Capacity of Prickly Pear Seeds. Acta Bot. Gall..

[88] Lumpuy-Castillo J., Amador-Martínez I., Díaz-Rojas M., Lorenzo O., Pedraza-Chaverri J., Sánchez-Lozada L.G., Aparicio-Trejo O.E. (2024). Role of Mitochondria in Reno-Cardiac Diseases: A Study of Bioenergetics, Biogenesis, and GSH Signaling in Disease Transition. Redox Biol..

[89] Li S., Li X., Rozanski G.J. (2003). Regulation of Glutathione in Cardiac Myocytes. J. Mol. Cell. Cardiol..

[90] Lash L.H. (2005). Role of Glutathione Transport Processes in Kidney Function. Toxicol. Appl. Pharmacol..

[91] Kumar P., Osahon O.W., Sekhar R.V. (2022). GlyNAC (Glycine and N-Acetylcysteine) Supplementation in Mice Increases Length of Life by Correcting Glutathione Deficiency, Oxidative Stress, Mitochondrial Dysfunction, Abnormalities in Mitophagy and Nutrient Sensing, and Genomic Damage. Nutrients.

[92] Zhang W., Deng J., Sunkara M., Morris A.J., Wang C., St Clair D., Vore M. (2015). Loss of Multidrug Resistance-Associated Protein 1 Potentiates Chronic Doxorubicin-Induced Cardiac Dysfunction in Mice. J. Pharmacol. Exp. Ther..

[93] Subramanyam D., Gurunathan D., Gaayathri R., Priya V.V. (2018). Comparative Evaluation of Salivary Malondialdehyde Levels as a Marker of Lipid Peroxidation in Early Childhood Caries. Eur. J. Dent..

[94] Soliman M.M., Alotaibi S.S., Sayed S., Hassan M.M., Althobaiti F., Aldhahrani A., Youssef G.B.A., El-Shehawi A.M. (2022). The Protective Impact of Salsola imbricata Leaf Extract from Taif Against Acrylamide-Induced Hepatic Inflammation and Oxidative Damage: The Role of Antioxidants, Cytokines, and Apoptosis-Associated Genes. Front. Vet. Sci..

[95] Scapagnini G., Sonya V., Nader A.G., Calogero C., Zella D., Fabio G. (2011). Modulation of Nrf2/ARE Pathway by Food Polyphenols: A Nutritional Neuroprotective Strategy for Cognitive and Neurodegenerative Disorders. Mol. Neurobiol..

[96] Ashrafizadeh M., Ahmadi Z., Samarghandian S., Mohammadinejad R., Yaribeygi H., Sathyapalan T., Sahebkar A. (2020). MicroRNA-Mediated Regulation of Nrf2 Signaling Pathway: Implications in Disease Therapy and Protection against Oxidative Stress. Life Sci..

[97] Bellezza I., Giambanco I., Minelli A., Donato R. (2018). Nrf2-Keap1 Signaling in Oxidative and Reductive Stress. Biochim. Biophys. Acta BBA-Mol. Cell Res..

[98] Gorrini C., Harris I.S., Mak T.W. (2013). Modulation of Oxidative Stress as an Anticancer Strategy. Nat. Rev. Drug Discov..

[99] Cengiz Z.T., Yılmaz H., Beyhan Y.E., Ekici A., Çiçek M., Aydemir S., Cengiz Z.T., Yılmaz H., Beyhan Y.E., Ekici A. (2023). The Importance of Antioxidant Enzymes and Oxidative Stress in Human Fascioliasis. Turk. Parazitol Derg..

[100] Dubois-Deruy E., Peugnet V., Turkieh A., Pinet F. (2020). Oxidative Stress in Cardiovascular Diseases. Antioxidants.

[101] Peoples J.N., Saraf A., Ghazal N., Pham T.T., Kwong J.Q. (2019). Mitochondrial Dysfunction and Oxidative Stress in Heart Disease. Exp. Mol. Med..

[102] Kim J.M., Kim H.G., Son C.G. (2018). Tissue-Specific Profiling of Oxidative Stress-Associated Transcriptome in a Healthy Mouse Model. Int. J. Mol. Sci..

[103] Gagour J., Ahmed M.N., Bouzid H.A., Oubannin S., Bijla L., Ibourki M., Hajib A., Koubachi J., Harhar H., Gharby S. (2022). Proximate Composition, Physicochemical, and Lipids Profiling and Elemental Profiling of Rapeseed (*Brassica napus* L.) and Sunflower (*Helianthus annuus* L.) Grown in Morocco. Evid.-Based Complement. Altern. Med..

[104] Minguez-Mosquera M.I., Gandul-Rojas B., Garrido-Fernandez J., Gallardo-Guerrero L. (1990). Pigments Present in Virgin Olive Oil. J. Am. Oil Chem. Soc..

[105] Espín J.C., Soler-Rivas C., Wichers H.J. (2000). Characterization of the Total Free Radical Scavenger Capacity of Vegetable Oils and Oil Fractions Using 2,2-Diphenyl-1-Picrylhydrazyl Radical. J. Agric. Food Chem..

[106] Dehpour A.A., Ebrahimzadeh M.A., Fazel N.S., Mohammad N.S. (2009). Antioxidant Activity of the Methanol Extract of *Ferula Assafoetida* and Its Essential Oil Composition. Grasas Aceites.

[107] Re R., Pellegrini N., Proteggente A., Pannala A., Yang M., Rice-Evans C. (1999). Antioxidant Activity Applying an Improved ABTS Radical Cation Decolorization Assay. Free Radic. Biol. Med..

[108] Huang B., Ke H., He J., Ban X., Zeng H., Wang Y. (2011). Extracts of Halenia Elliptica Exhibit Antioxidant Properties in Vitro and in Vivo. Food Chem. Toxicol. Int. J. Publ. Br. Ind. Biol. Res. Assoc..

[109] Oyaizu M., Oyaizu M. (1986). Studies on Products of Browning Reaction Antioxidative Activities of Products of Browning Reaction Prepared from Glucosamine. Jpn. J. Nutr..

[110] Masocha W. (2009). Systemic lipopolysaccharide (LPS)-Induced Microglial Activation Results in Different Temporal Reduction of CD200 and CD200 Receptor Gene Expression in the Brain. J. Neuroimmunol..

[111] Qin L., Wu X., Block M.L., Liu Y., Breese G.R., Hong J.-S., Knapp D.J., Crews F.T. (2007). Systemic LPS Causes Chronic Neuroinflammation and Progressive Neurodegeneration. Glia.

[112] Beyer W.F., Fridovich I. (1987). Assaying for Superoxide Dismutase Activity: Some Large Consequences of Minor Changes in Conditions. Anal. Biochem..

[113] Beers R.F., Sizer I.W. (1952). A Spectrophotometric Method for Measuring the Breakdown of Hydrogen Peroxide by Catalase. J. Biol. Chem..

[114] Mills G.C. (1959). The Purification and Properties of Glutathione Peroxidase of Erythrocytes. J. Biol. Chem..

[115] Ellman G.L. (1959). Tissue Sulfhydryl Groups. Arch. Biochem. Biophys..

[116] Ohkawa H., Ohishi N., Yagi K. (1979). Assay for Lipid Peroxides in Animal Tissues by Thiobarbituric Acid Reaction. Anal. Biochem..

