# Supplementary material for: Protective Antioxidant Potential of Argan Oil Versus Other Edible Oils in LPS-Challenged Mouse Heart and Kidney
Source: Int J Mol Sci. 2025 Aug 27;26(17):8300. doi: 10.3390/ijms26178300 (PMC12428758; doi:10.3390/ijms26178300)
Supplement: Supplementary file 1 [file ijms-26-08300-s001.zip › ijms-3783070-supplementary.pdf]

Supplementary Table S1. Pearson correlation between oxidative stress parameters in heart and kidney of mice (*** $p \leq 0.001$, ** $p \leq 0.01$, * $p \leq 0.05$).

Pearson correlation	SOD		CAT		GPx		GSH		MDA	
	r	p-value	r	p-value	r	p-value	r	p-value	r	p-value
**Heart**										
**SOD**	1	0	0.80*	0.005	0.75*	0.012	-0.31	0.369	0.84*	0.002
**CAT**			1	0	0.82*	0.003	-0.34	0.326	0.94***	≤0.001
**GPx**					1	0	-0.48	0.152	0.80*	0.005
**GSH**							1	0	-0.42	0.225
**MDA**									1	0
**Kidney**										
**SOD**	1	0	0.95***	≤0.001	0.88**	0.001	0.63	0.051	0.73*	0.018
**CAT**			1	0	0.90***	≤0.001	0.60	0.065	0.72*	0.019
**GPx**					1	0	0.53	0.119	0.90***	≤0.001
**GSH**							1	0	0.24	0.509
**MDA**									1	0
